# Strebluses E–H, four new stilbene-like derivatives from the stems of *Streblus ilicifolius*†

**DOI:** 10.1039/d2ra07294g

**Published:** 2022-12-22

**Authors:** Tho Huu Le, Phu Hoang Dang, Hai Xuan Nguyen, Truong Nhat Van Do, Nhan Trung Nguyen, Mai Thanh Thi Nguyen

**Affiliations:** Faculty of Chemistry, University of Science Ho Chi Minh City 72711 Vietnam; Vietnam National University Ho Chi Minh City 71300 Vietnam nttmai@hcmus.edu.vn ntnhan@hcmus.edu.vn; Research Lab for Drug Discovery and Development, University of Science Ho Chi Minh City 72711 Vietnam

## Abstract

Following bioactivity-guided isolation, four new stilbene-like derivatives, named Strebluses E–H, were isolated from the EtOAc-soluble fraction of the stems of *Streblus ilicifolius* (Moraceae). Their chemical structures were elucidated based on NMR spectroscopic data interpretation and optical rotation calculation. *Streblus* E possesses potent tyrosinase inhibitory activity with an IC_50_ value of 0.1 μM. Oxy-tyrosinase has two bound Cu^2+^ ions and a peroxide group in the binding site, which has a role in the catalytic oxidation. Thus, a docking study of *Streblus* E with oxy-tyrosinase was performed to analyze the ligand–protein interactions. With *in silico* modelling, the *S* value and the ligand–protein interactions suggested that *Streblus* E showed lower binding affinity for oxy-tyrosinase than that of *Streblus* C.

## Abstract 2

*Streblus* E was a potent tyrosinase inhibitor isolated from the stems of *Streblus ilicifolius*. Its docking study with oxy-tyrosinase was carried out to clarify their interactive model.

## Graphical abstract

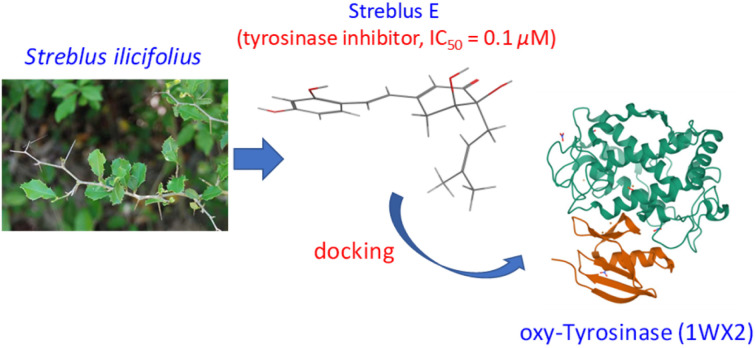

## Introduction

*Streblus ilicifolius* (S. Vidal) Corn. (Moraceae) is widely distributed in India, China, and South Asia.^1^ Several phytochemical studies of *S. ilicifolius* have been carried out, leading to the identification of amide glycosides, coumarins, stilbenes, lignans, and polyphenols. In addition, the anti-tyrosinase, antimicrobial, and anti-inflammatory activities of these chemical constituents has been reported.^2–4^

Our continued studies on the bioactivity-guided phytochemical investigation of medicinal plants for tyrosinase inhibitory activity^5–9^ has led to the identification of two coumarins (Strebluses A and B) and two stilbene-like derivatives (Strebluses C and D) from the stems of *Streblus ilicifolius*, among which *Streblus* C exhibited a remarkable inhibitory effect with an IC_50_ value of 0.01 μM.^10,11^ Thus, this phytochemical study was continuously performed, leading to the isolation of four new stilbene-like derivatives, Strebluses E–H (1–4). Their structures were determined by NMR spectroscopic interpretation. In addition, their absolute configurations were identified based on the optical rotation calculation. Herein, the tyrosinase inhibitory activity assays and the molecular docking studies with the oxy-tyrosinase were performed.

## Results and discussion

### Structural elucidation of four new isolated compounds from *S. ilicifolius*

**Fig. 1:**
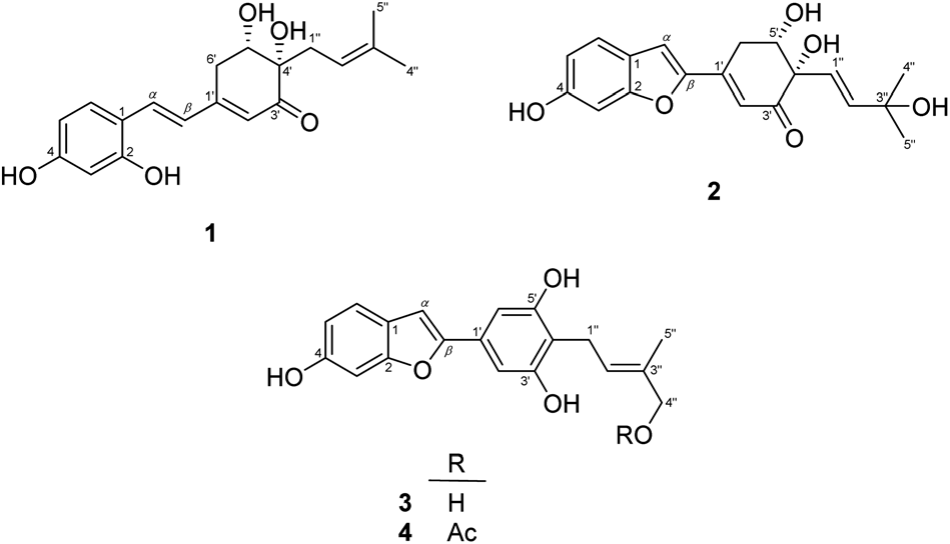
The structure of compounds 1–4.

The EtOAc-soluble fraction of *S. ilicifolius* stems was chromatographed to obtain four undescribed stilbene-like derivatives, Strebluses E–H (1–4) (Fig. 1).

**Fig. 2:**
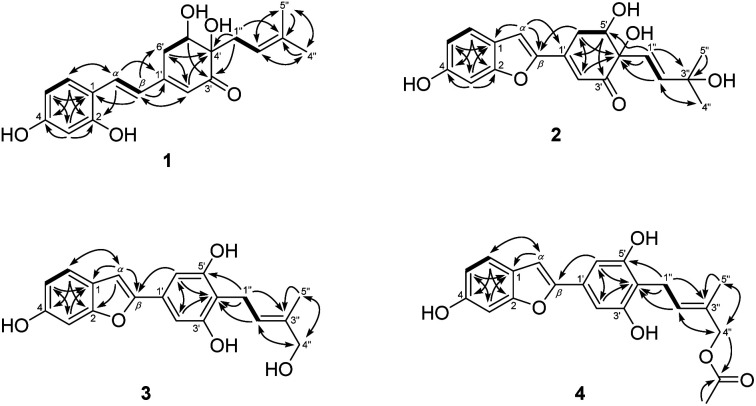
^1^H–^1^H COSY (bold lines) and HMBC (solid arrows) correlations observed for 1–4.

**Fig. 3:**
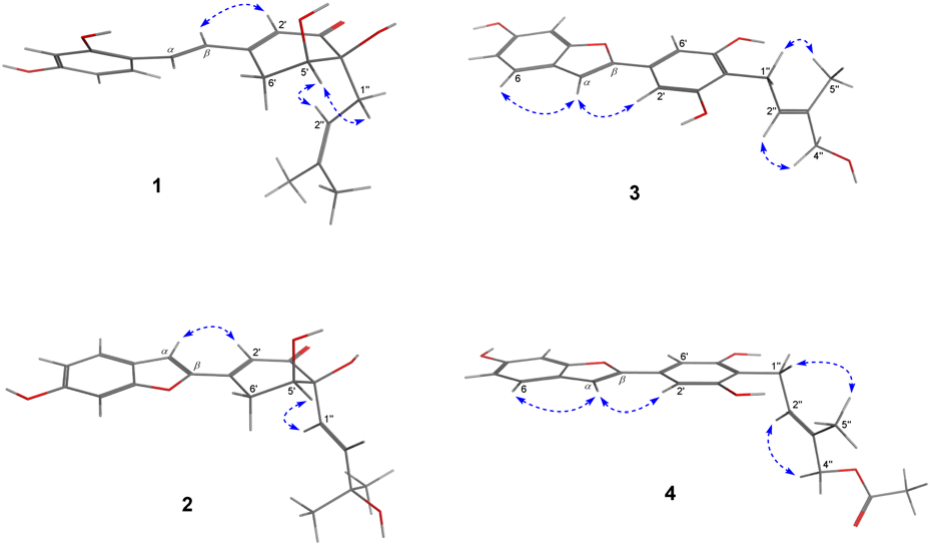
NOESY correlations observed for 1–4.

Compound 1, *Streblus* E, showed a molecular formula to be C_19_H_22_O_5_ based on the quasi-molecular ion at *m*/*z* 331.1545 [M + H]^+^ (calcd for C_19_H_23_O_5_^+^, 331.1540) in the HRESIMS spectrum. The ^1^H and ^13^C NMR spectra showed signals of the prenylated stilbene-like feature having the 5,6-dihydroxycyclohex-2-en-1-one moiety (Tables 1 and 2), which resembled those of *Streblus* C except for the absence of the acetonide group.^10^ The observed HMBC correlations (Fig. 2) indicated the presence of the 2,4-dihydroxyphenyl group in 1. The 5,6-dihydroxycyclohex-2-en-1-one substructure was established based on the HMBC correlations from H-2′ to C-4′ and C-6′, from H-5′ to C-1′ and C-3′, from 5′-OH to C-5′ and C-6′, and from 4′-OH to C-3′ and C-4′. The HMBC correlations from H-*α* to C-2, C-6, and C-1′, from H-*β* to C-1, C-1′, C-2′, and C-6′ were supportive of the C*α*–C1 and C*β*–C1′ bonds. In addition, the C-4′ prenyl group was assigned based on the HMBC correlations. The NOESY correlation between H-5′/H_2_-1′′/H-2′′ deduced the presence of the *cis*-diol (Fig. 3). In addition, the ^3^*J* coupling constants between H-5′ and H_2_-6′ were 5.6 and 3.2 Hz, to suggest the equatorial configuration of H-5′.^12^ The preferred conformers of *cis*-(*R*,*R*)-diol 1 were established by molecular mechanics calculation using MMFF94 force field.^13^ The obtained conformers were reoptimized using B3LYP density functional theory (DFT) method with the 6-31G* basis set, to obtain the most preferred conformer with 95.5% Boltzmann distribution. The optical rotation calculation at the sodium D line (*λ* = 589.3 nm) was carried out using DFT-B3LYP/6-311++G(2d,2p) function with the polarizable continuum model (PCM) for methanol.^14,15^ The calculated [*α*]_D_ value of *cis*-(*R*,*R*)-diol 1 was obtained as −694.12, while the experimental value of [*α*]_D_ +630.0 (*c* 0.01, MeOH) was the opposite in sign. Thus, an (*S*,*S*) absolute configuration was concluded for *Streblus* E (1), which was deacetonide-*ent-Streblus* C.

**Table 1:** ^1^H (500 MHz) NMR data (acetone-*d*_6_) for compounds 1–4

Position	*δ*_H_ (*J*, Hz)			
	1	2	3	4
3	6.46, d (2.4)	7.00, d (2.1)	6.96, d (2.2)	6.95, d (2.0)
5	6.42, dd (8.5, 2.4)	6.87, dd (8.5, 2.1)	6.80, dd (8.4, 2.2)	6.80, dd (8.4, 2.0)
6	7.47, d (8.5)	7.52, d (8.5)	7.38, d (8.4)	7.38, d (8.4)
2′	5.97, brs	6.55, d (2.3)	6.92, s	6.92, s
5′	4.23, dd (5.6, 3.2)	4.15, dd (2.9, 2.5)		
6′	2.94–2.96, m	3.15, ddd (18.3, 2.9, 2.3)	6.92, s	6.92, s
		3.08, dd (18.3, 2.5)		
*α*	7.36, d (16.3)	7.33, s	6.91, brs	6.91, brs
*β*	7.00, d (16.3)			
1′′	2.42, dd (14.7, 7.6)	5.91, d (15.5)	3.43, d (7.3)	3.45, d (7.3)
	2.34, dd (14.7, 6.9)			
2′′	5.19, dd (7.6, 6.9)	6.10, d (15.5)	5.57, tq (7.3, 1.3)	5.66, tq (7.3, 1.3)
4′′	1.67, s	1.22, s	3.90, d (6.2)	4.41, s
5′′	1.57, s	1.19, s	1.80, d (1.3)	1.82, d (1.3)
2-OH	8.91, s			
4-OH	8.68, s	8.93, s	8.46, s	8.46, s
4′-OH	4.10, s	4.37, s		
5′-OH	3.65, s	4.00, s	8.33, s	8.42, s
3′′-OH		3.62, s		
3′-OH			8.33, s	8.42, s
4′′-OH			3.58, t (6.2)	
4′′-OAc				1.98, s

**Table 2:** ^13^C (125 MHz) NMR data (acetone-*d*_6_) for compounds 1–4

Position	*δ*_C_, type C			
	1	2	3	4
1	116.3	122.0	122.7	122.7
2	158.2	158.1	156.7	156.7
3	103.7	98.3	98.4	98.4
4	160.8	158.9	156.6	156.6
5	109.0	114.3	113.2	113.2
6	129.5	123.4	121.8	121.9
1′	154.9	144.2	130.0	130.2
2′	123.0	119.0	103.8	103.9
3′	201.1	199.1	157.3	157.3
4′	79.7	80.3	116.1	115.3
5′	72.9	74.5	157.3	157.3
6′	31.9	32.1	103.9	103.9
*α*	132.1	111.0	101.5	101.6
*β*	126.4	153.2	155.8	155.7
1′′	35.5	124.6	22.7	22.8
2′′	119.0	142.4	123.9	128.5
3′′	134.8	70.3	135.7	130.6
4′′	26.1	30.4	68.6	70.5
5′′	18.1	30.2	13.9	14.1
4′′-OCOC̲H_3_			20.8	
4′′-OC̲OCH_3_			170.8	

Compound 2, *Streblus* F, showed the sodium adduct molecular ion at *m*/*z* 367.1169 [M + Na]^+^ (calcd for C_19_H_20_O_6_Na^+^, 367.1152) in the HRESIMS spectrum. The ^1^H and ^13^C NMR spectra of 2 closely resembled those of *Streblus* D, except for the presence of the 3-hydroxyisopent-1(*E*)-enyl group [*δ*_H_ 5.91 (d, *J* = 15.5 Hz, H-1′′), 6.10 (d, *J* = 15.5 Hz, H-2′′), 1.22 and 1.19 (s, 2 × 3′′-CH_3_)] instead of the prenyl group in *Streblus* D (Tables 1 and 2). The observed NOESY correlation between H-5′ and H-1′′ confirmed the *cis*-orientation of the C-4′ and C-5′ hydroxy groups (Fig. 3). In addition, the equatorial configuration of H-5′ was suggested based on its ^3^*J* coupling constants of 2.9 and 2.5 Hz.^12^ The conformational analysis for (*R*,*R*)-2 was obtained seven conformers with a total Boltzmann weight >99%. The calculated [*α*]_D_ value of (*R*,*R*)-2 was +127.95, opposite in sign to its experimental value [*α*]_D_ −134.0 (*c* 0.01, MeOH). Thus, the structure of *Streblus* F (2) was concluded as 4′*S*,5′*S*.

*Streblus* G (3) showed the molecular formula C_19_H_18_O_5_, as deduced from the negative HRESIMS spectrum at *m*/*z* 325.1087 [M − H]^−^ (calcd for C_19_H_17_O_5_^−^, 325.1081). The ^1^H spectrum showed signals for a 1,3,4-trisubstituted [*δ*_H_ 7.38 (d, *J* = 8.4 Hz, H-6), 6.96 (d, *J* = 2.2 Hz, H-3), 6.80 (dd, *J* = 8.4, 2.2 Hz, H-5)] and a 1,3,4,5-tetrasubstituted [*δ*_H_ 6.92 (s, H-2′ and H-6′)] aromatic rings, an olefinic proton [*δ*_H_ 6.91 (brs, H-*α*)], a hydroxylated prenyl group [*δ*_H_ 3.43 (d, *J* = 7.3 Hz, H_2_-1′′), 5.57 (tq, *J* = 7.3, 1.3 Hz, H-2′′), 3.90 (d, *J* = 6.2 Hz, H_2_-4′′), 1.80 (d, *J* = 1.3 Hz, H_3_-5′′), 3.58 (t, *J* = 6.2 Hz, 4′′-OH)], and two hydroxy groups [*δ*_H_ 8.46 (s, 6-OH), 8.33 (s, 3′-OH and 5′-OH)]. The ^13^C NMR data exhibited resonances for 14 aromatic carbons [*δ*_C_ 98.3–157.2] and a hydroxylated prenyl group [*δ*_C_ 135.5 (C-3′′), 123.7 (C-2′′), 68.5 (C-4′′), 22.5 (C-1′′), 13.7 (C-5′′)] (Table 2). These data resembled closely those of moracin M, except for the presence of the hydroxylated prenyl group at C-4′.^16^ The HMBC correlations from H-6 to C-*α*, C-4, and C-2, from H-5 to C-1 and C-3, from H-3 to C-1 and C-5, from H-*α* to C-*β*, C-1, and C-2, from H-2′/H-6′ to C-*β* permitted the structural assignment of 3 as shown (Fig. 2). The C-3′ and C-5′ hydroxy groups were identified by the HMBC correlations with two corresponding oxygenated aromatic carbons. In addition, the HMBC correlations from H_2_-1′′ and H-2′′ to C-4′, from H-2′′ and H_3_-5′′ to C-4′′ indicated the location of the 4′′-hydroxyprenyl group at C-4′. The NOESY correlations between H-6/H-*α*/H-2′(6′), H_2_-1′′/H_3_-5′′, and H-2′′/H_2_-4′′ confirmed the presence of the 2-phenylbenzofuran moiety and the (2′′*E*)-4′′-hydroxyprenyl group (Fig. 3). Thus, the structure of *Streblus* G (3) was concluded as 4′-(4′′-hydroxyprenyl)moracin M.

*Streblus* H (4) showed a sodiated molecular ion peak at *m*/*z* 391.1151 [M + Na]^+^ (calcd for C_21_H_20_O_6_Na, 391.1152) in the HRESIMS spectrum. The ^1^H and ^13^C NMR spectra of 4 resembled closely those of 3, except for the presence of signals for an acetyl group [*δ*_H_ 2.08; *δ*_C_ 170.8, 20.8] (Table 2). The HMBC correlation between the H_2_-4′′ oxymethylene protons and the acetoxy carbonyl at *δ*_C_ 170.8 suggested that the acetylation happened at C-4′′ (Fig. 2). In addition, the NOESY correlations between H_2_-1′′/H_3_-5′′ and H-2′′/H_2_-4′′ supported the *E* configuration of the double bond in 4′′-acetoxyprenyl group (Fig. 3). Thus, the structure of *Streblus* H (4) was assigned as 4′′-acetyl*streblus* G.

### Tyrosinase inhibitory activity and docking studies

**Fig. 4:**
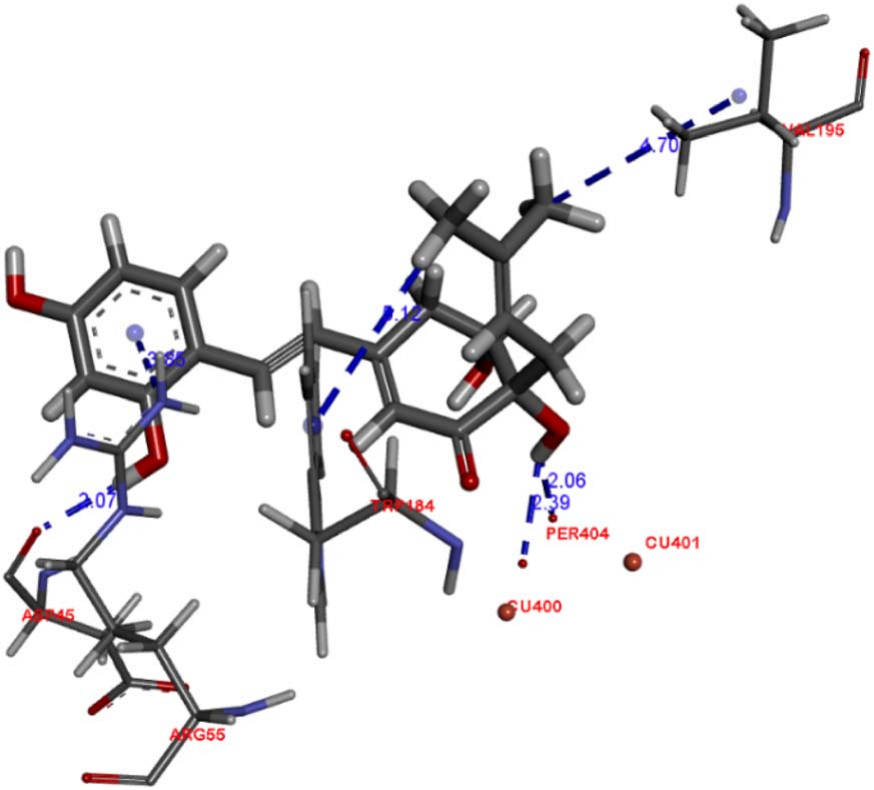
Docked pose of best ranked docking score of compound 1.

All isolated compounds were tested for their tyrosinase inhibitory activities.^17^ Kojic acid was used as a positive control. *Streblus* E (1) showed potent inhibitory effect with an IC_50_ value of 0.1 μM as compared to that of kojic acid (IC_50_, 44.6 μM). All remaining compounds were inactive (IC_50_ > 100 μM). The docking study of 1 was performed with MOE following our previous procedure.^9^ In the binding pocket of the top-rank pose, 1 showed the H-donor interaction from the C-4′ hydroxy group to peroxide bridge PER404, and from the C-2 hydroxy group to ASP45 residue (Fig. 4). The aromatic ring had the π-cation interaction with ARG55 residue. In addition, the prenyl group showed the σ–σ and π–σ interactions with VAL195 and TRP184, respectively.

The genus *Streblus* is a small deciduous shrub, which includes 20 species and mainly distributed in South China and South Asia. The phytochemical studies of *Streblus ilicifolius* were carried out to obtain various class of compounds. In our previous studies, we reported the structure and the anti-tyrosinase evaluation of four new compounds, in which *Streblus* C showed noteworthy inhibitory activity.^10,11^ With these interesting results, we continued to carry out the bioactivity-guided isolation, leading to the identification of four new stilbene-like derivatives were isolated as well as their *in vitro* and *in silico* tyrosinase inhibitory activity were reported. In this study, *Streblus* E (1) showed potent inhibitory effect with an IC_50_ value of 0.1 μM. The 2,4-resorcinol subunit highly contributed to inhibitory activity.^18^ In addition, the formation of the five-membered ring gave rise to the loss of inhibitory effect.^19^ With *in silico* modelling, the *S* value and the ligand–protein interactions suggested that 1 showed lower binding affinity for oxy-tyrosinase than that of S*treblus* C.^10^ This result was used to clarify the remarkable difference in IC_50_ values of Strebluses C (0.01 μM) and E (0.1 μM).

## Conclusions

Four new stilbene-like derivatives, named Strebluses E–H (1–4), were isolated from the EtOAc-soluble fraction of the stems of *Streblus ilicifolius* (Moraceae). Their structures were elucidated based on NMR spectroscopic data interpretation with the aid of optical rotation calculation. *Streblus* E (1) showed potent tyrosinase inhibitory effect with an IC_50_ value of 0.1 μM.

## Experimental

### General experimental procedures

Optical values were measured on a Shimadzu UV-1800 spectrophotometer (Shimadzu Pte., Ltd., Singapore). NMR spectra were acquired on a Bruker Avance III 500 spectrometer (Bruker BioSpin AG, Bangkok, Thailand). Chemical shifts are expressed as *δ* values. HRESIMS data were acquired on Bruker micrOTOF-QII mass spectrometer (Bruker Singapore Pte., Ltd., Singapore). Column chromatography (CC) was carried out using silica gel 60, 0.06–0.2 mm (Scharlau, Barcelona, Spain) and LiChroprep RP-18, 40–63 μm (Merck KGaA, Darmstadt, Germany). Kieselgel 60 F_254_ or RP-18 F_254_ plates for thin-layer chromatography (TLC) were purchased from Merck (Merck KGaA, Darmstadt, Germany). Mushroom tyrosinase (EC 1.14.18.1; 3933 U mL^−1^) and l-dihydroxyphenylalanine (l-DOPA) were obtained from Sigma-Aldrich (Sigma-Aldrich Pte Ltd, Singapore). Other chemicals were of the highest grade available.

### Plant material

The stems of *Streblus ilicifolius* were collected at Hoai Nhon District, Binh Dinh Province, Vietnam, in October 2017. Its scientific name was identified by Dr Rer. Nat. Anh Tuan Dang-Le, Faculty of Biology and Biotechnology, University of Science, Ho Chi Minh City, Vietnam. A sample (MCE0052) has been deposited at the Department of Medicinal Chemistry, Faculty of Chemistry, University of Science, Ho Chi Minh City, Vietnam.

### Extraction and isolation

The dried powdered stems of *S. ilicifolius* (7.0 kg) were exhaustively extracted in a Soxhlet extractor with *n*-hexane, EtOAc, and MeOH to yield *n*-hexane – (64.8 g), EtOAc – (117.2 g), and MeOH – (378.0 g) soluble fractions, respectively. The EtOAc-soluble fraction was chromatographed by silica gel CC (15 × 150 cm) and eluted with MeOH–CHCl_3_ mixtures (v/v, 0 : 100 → 100 : 0) to afford 18 fractions (Fr.1–Fr.18). Fraction Fr.8 (0.8 g) was subjected to further silica gel CC and was eluted with MeOH–CHCl_3_ (v/v, 0 : 100 → 100 : 0) mixtures to yield 2 fraction (Fr.8.1 and Fr.8.2). Fraction Fr.8.1 (445 mg) was chromatographed over a silica gel column with EtOAc–CHCl_3_ mixtures (v/v, 0 : 100 → 100 : 0) mixtures to obtain five fractions (Fr.8.1.1 and Fr.8.1.5). Fraction Fr.8.1.4 (50.9 mg) was purified by preparative TLC with an EtOAc–CHCl_3_ mixture (v/v, 30 : 70) to afford compound 2 (3.0 mg). Fraction Fr.11 (4.7 g) was subjected to silica gel CC with MeOH–CHCl_3_ mixtures (v/v, 0 : 100 → 100 : 0) to obtain six fractions (Fr.11.1–Fr.11.6). Fraction Fr.11.5 (849 mg) was separated by silica gel CC with EtOAc–CHCl_3_ mixtures (v/v, 0 : 100 → 100 : 0) to obtain four fractions (Fr.11.5.1–Fr.11.5.4). Fraction Fr.11.5.2 (118 mg) was purified by CC with Me_2_CO–*n*-hexane mixtures (v/v, 0 : 100 → 100 : 0), then the resulting fraction was purified by preparative TLC with an EtOAc–*n*-hexane mixture (v/v, 50 : 50) to afford compound 4 (4.0 mg). Fraction Fr.14 (19.6 g) was separated by silica gel CC with MeOH–CHCl_3_ mixtures (v/v, 0 : 100 → 100 : 0) to obtain 11 fractions (Fr.14.1–Fr.14.11). Fraction Fr.14.9 (8.8 g) was loaded onto a silica gel column and eluted with Me_2_CO–CHCl_3_ mixtures (v/v, 0 : 100 → 70 : 30) to give 20 fractions (Fr.14.9.1–Fr.14.9.20). Fraction Fr.14.9.5 (41.4 mg) was chromatographed over a silica gel column with a MeOH–CHCl_3_ mixture (v/v, 10 : 90) to afford compound 3 (4.0 mg). Fraction Fr.14.9.8 (46.8 mg) was separated by CC with Me_2_CO–CHCl_3_ mixtures (v/v, 0 : 100 → 70 : 30), then the resulting fractions were purified by preparative reversed-phase TLC with a H_2_O–MeOH mixture (v/v, 30 : 70) to afford compound 1 (3.0 mg).

*Streblus* E (1): yellow, amorphous powder; ^1^H and ^13^C NMR (500 MHz, acetone-*d*_6_, see Tables 1 and 2); HRESIMS *m*/*z* 331.1545 [M + H]^+^ (calcd for C_19_H_23_O_5_^+^, 331.1540).

*Streblus* F (2): yellow, amorphous powder; ^1^H and ^13^C NMR (500 MHz, acetone-*d*_6_, see Tables 1 and 2); HRESIMS *m*/*z* 367.1169 [M + Na]^+^ (calcd for C_19_H_20_O_6_Na^+^, 367.1152).

*Streblus* G (3): yellow, amorphous powder; ^1^H and ^13^C NMR (500 MHz, acetone-*d*_6_, see Tables 1 and 2); HRESIMS *m*/*z* 325.1087 [M − H]^−^ (calcd for C_19_H_17_O_5_^−^, 325.1081).

*Streblus* H (4): yellow, amorphous powder; ^1^H and ^13^C NMR (500 MHz, acetone-*d*_6_, see Tables 1 and 2); HRESIMS *m*/*z* 391.1151 [M + Na]^+^ (calcd for C_21_H_20_O_6_Na^+^, 391.1152).

### Tyrosinase inhibitory assay

All pure compounds were dissolved in DMSO and tested at concentrations of 0.01–100 μM. Assay mixtures in 0.1 M phosphate buffer pH 6.8 were prepared immediately before use, consisting of 100 μL of tyrosinase solution (15 U mL^−1^) and 1900 μL of test solution. These mixtures were preincubated at 32 °C for 30 min, followed by 1000 μL of l-DOPA 1.5 mM in pH 6.8 phosphate buffer, and incubated at 32 °C for 7 min. The absorbance (*A*) at 475 nm was acquired on Shimadzu UV-1800 spectrophotometer. The inhibitory percentage (*I*%) was calculated according to the formula: *I*% = [(*A*_control_ − *A*_sample_)/*A*_control_] × 100%. All experiments were performed in triplicate and data were represented as the mean of three samples with standard deviation.

### Optical rotation calculation

The conformational searches were performed on Spartan'18 (Wave function, Inc., Irvine, USA) using Merck molecular force field (MMFF). All conformers with Boltzmann weight > 1% were optimized using DFT method at the B3LYP/6-31G* level in the gas phase. The optical rotation calculations at 589.3 nm were carried out using the DFT-B3LYP with 6-311++G(2d,2p) basis set in IEFPCM solvation model for methanol. These calculations were performed on Gaussian 16 Rev. C.01 (Gaussian, Inc., Wallingford, USA). The calculated optical rotation values were expressed as the Boltzmann-weighted average of all output data.

### Molecular docking

Docking studies were performed with Molecular Operating Environment 2019 (MOE 2019.0102) suite (Chemical Computing Group ULC, Montreal, Canada). All structures were minimized up to 0.0001 gradients using the Amber12:EHT force field. The oxy-tyrosinase structure (1WX2) was prepared following our previous procedure. The molecular docking procedure was carried out with Triangle Matcher placement, Induced Fit refinement, and two scoring methods (London dG and GBVI/WSA dG). Five top results with the negative binding free energy value (*S* value) were selected to show up. The ligand interactions were carried out using BIOVIA Discovery Studio Visualizer 2016 (Dassault Systèmes Americas Corp., Waltham, USA).

## Author contributions

MTTN and NTN designed the work. THL, HXN, and TNVD directed the experiments. THL and TNVD performed tyrosinase inhibitory assay. PHD performed molecular docking and optical rotation calculations. The manuscript was written by THL, PHD, MTTN, and NTN.

## Conflicts of interest

There are no conflicts to declare.

## Supplementary Material

RA-013-D2RA07294G-s001
