# Supplementary material for: Strebluses E–H, four new stilbene-like derivatives from the stems of Streblus ilicifolius
Source: RSC Adv. 2022 Dec 22;13(1):570–4. doi: 10.1039/d2ra07294g (PMC9773016; doi:10.1039/d2ra07294g)
Supplement: RA-013-D2RA07294G-s001 [file RA-013-D2RA07294G-s001.pdf]

Electronic Supplementary Material (ESI) for RSC Advances.
This journal is © The Royal Society of Chemistry 2022

Supplementary data

Strebluses E–H, four new stilbene-like derivatives from the stems of *Streblus ilicifolius*

Tho Huu Le, [abc] Phu Hoang Dang, [abc] Hai Xuan Nguyen, [abc] Truong Nhat Van Do, [abc] Nhan Trung Nguyen, *[abc] and Mai Thanh Thi Nguyen *[abc]

[a] *Faculty of Chemistry, University of Science, Ho Chi Minh City, 72711, Vietnam.*
E-mail: nttmai@hcmus.edu.vn
Email: ntnhan@hcmus.edu.vn
[b] *Vietnam National University, Ho Chi Minh City, 71300, Vietnam*
[c] *Research Lab for Drug Discovery and Development, University of Science, Ho Chi Minh City, 72711, Vietnam*

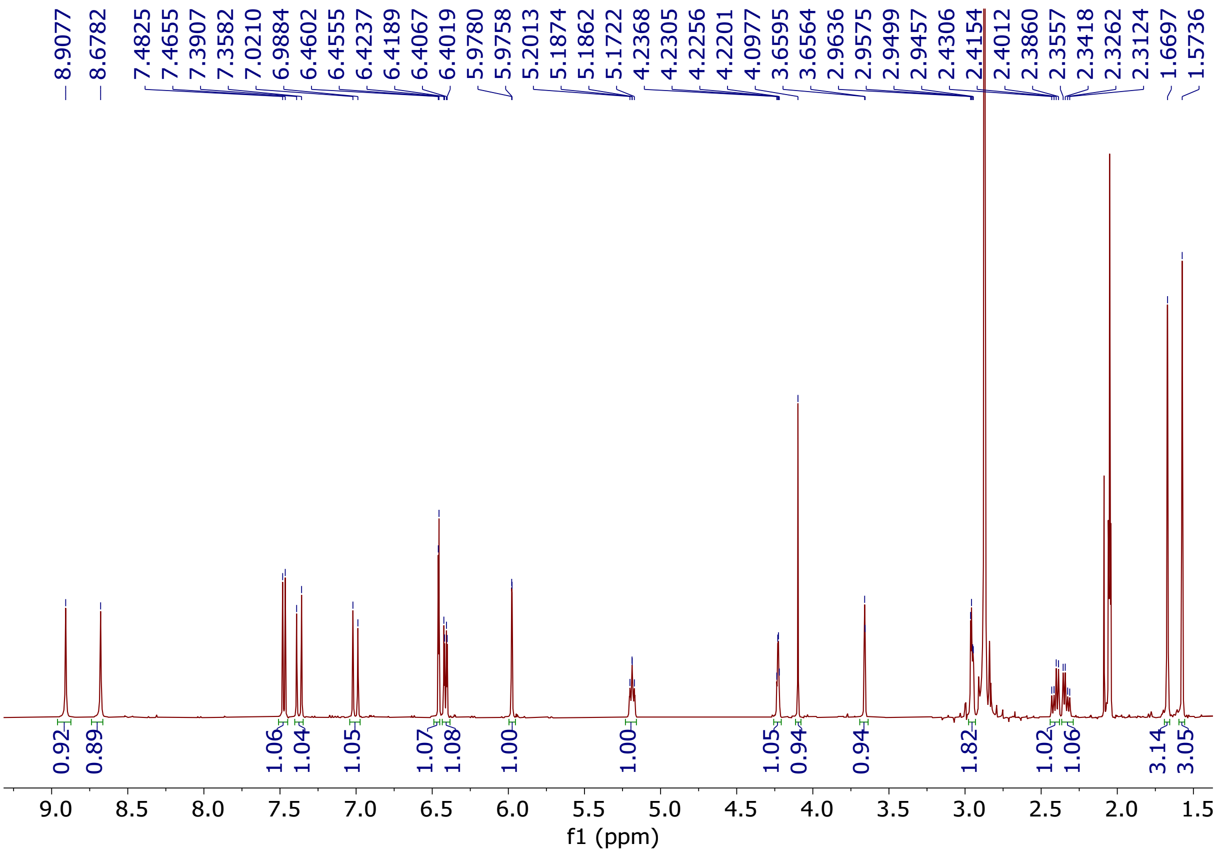

Figure S1. ^{1}H NMR spectrum of **1** (500 MHz, acetone-d_6).

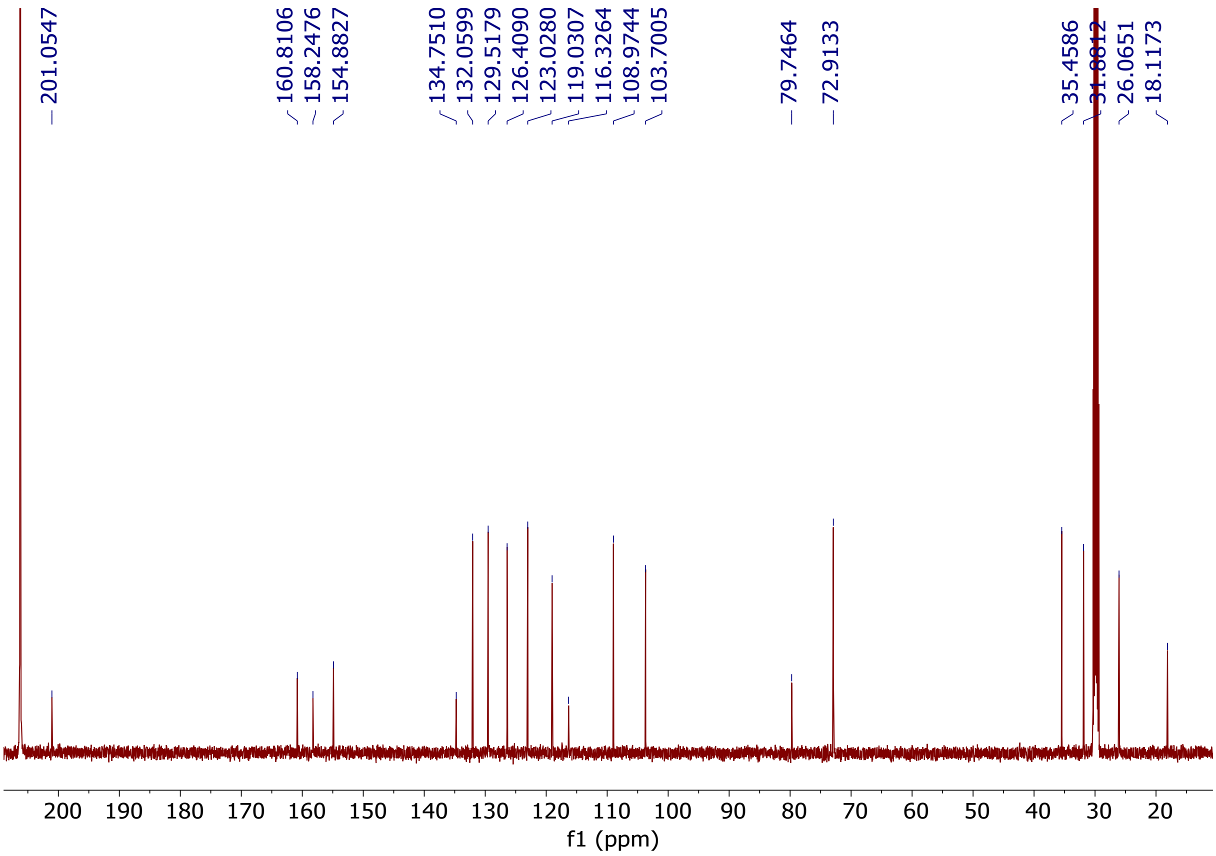

Figure S2. ^{13}C NMR spectrum of **1** (125 MHz, acetone-d_6).

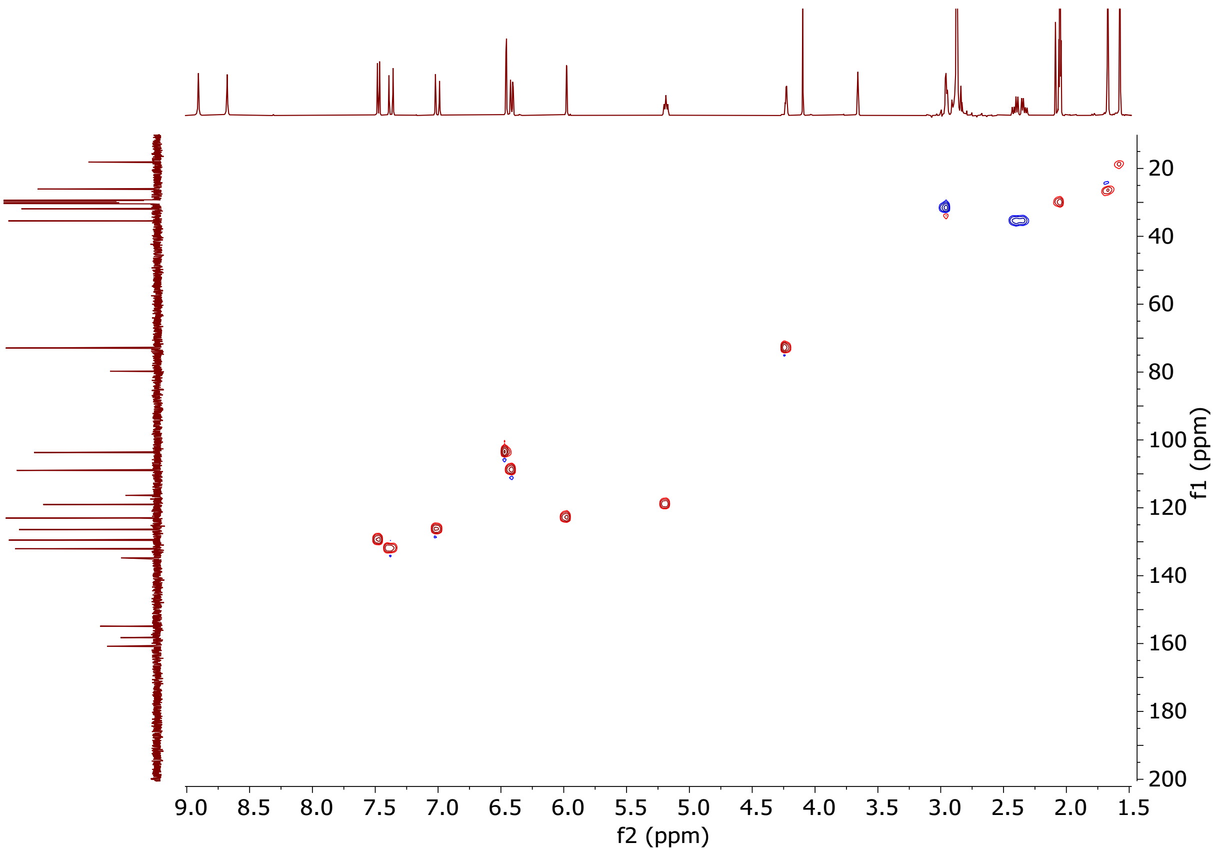

Figure S3. HSQC NMR spectrum of **1**.

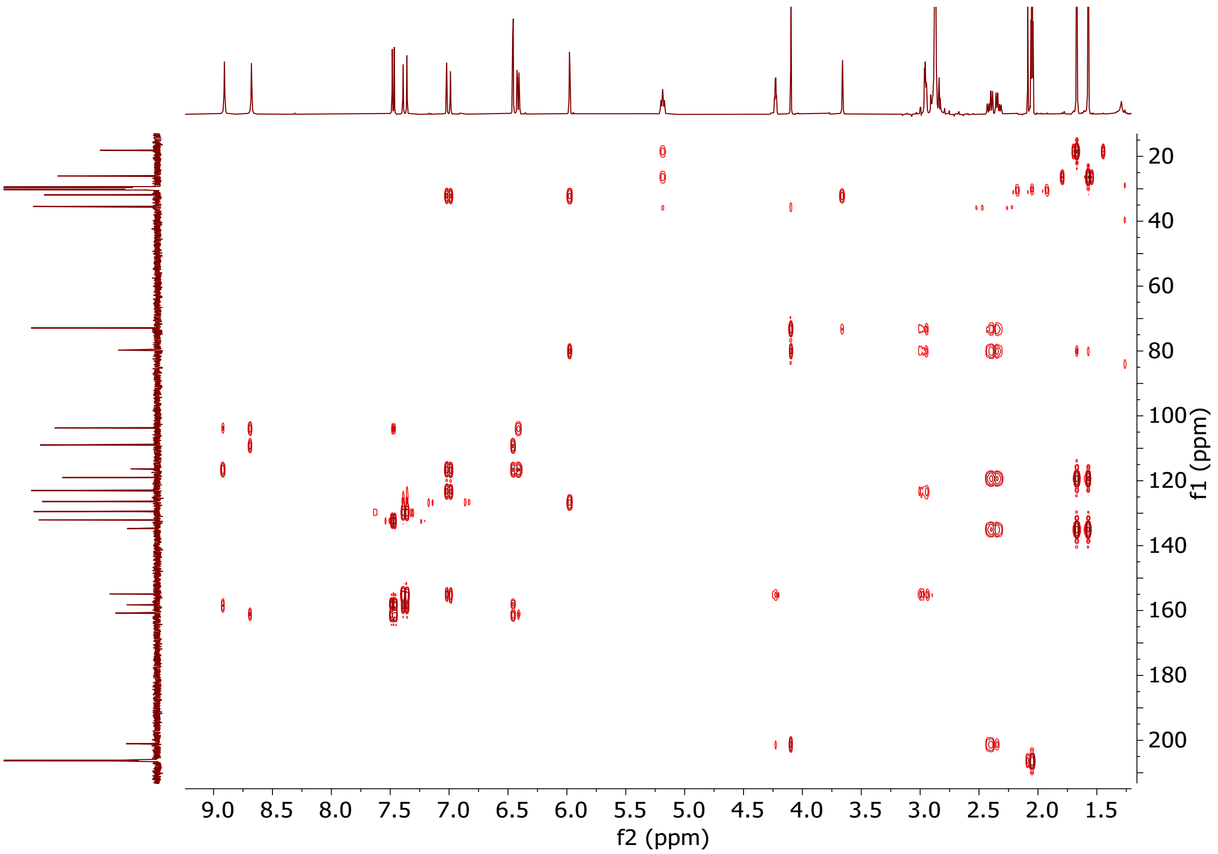

Figure S4. HMBC NMR spectrum of **1**.

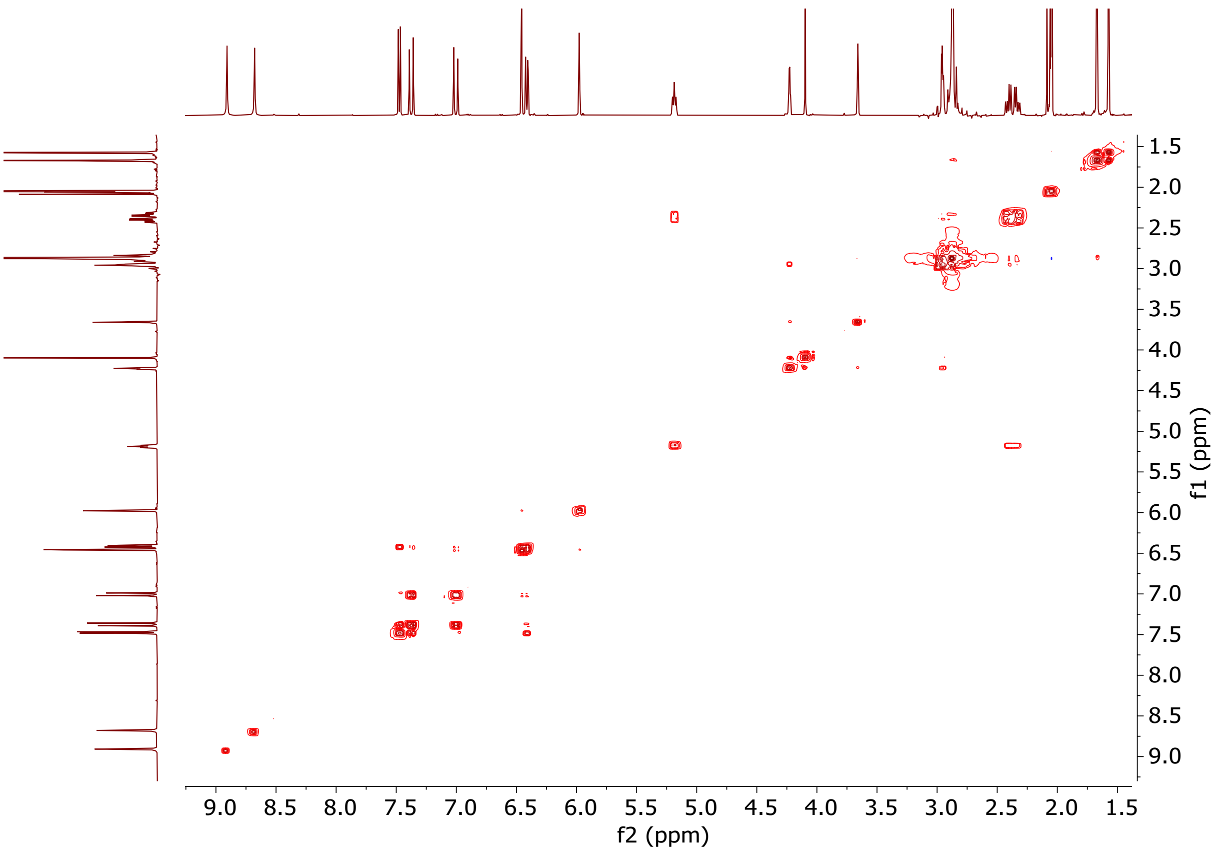

Figure S5. COSY NMR spectrum of **1**.

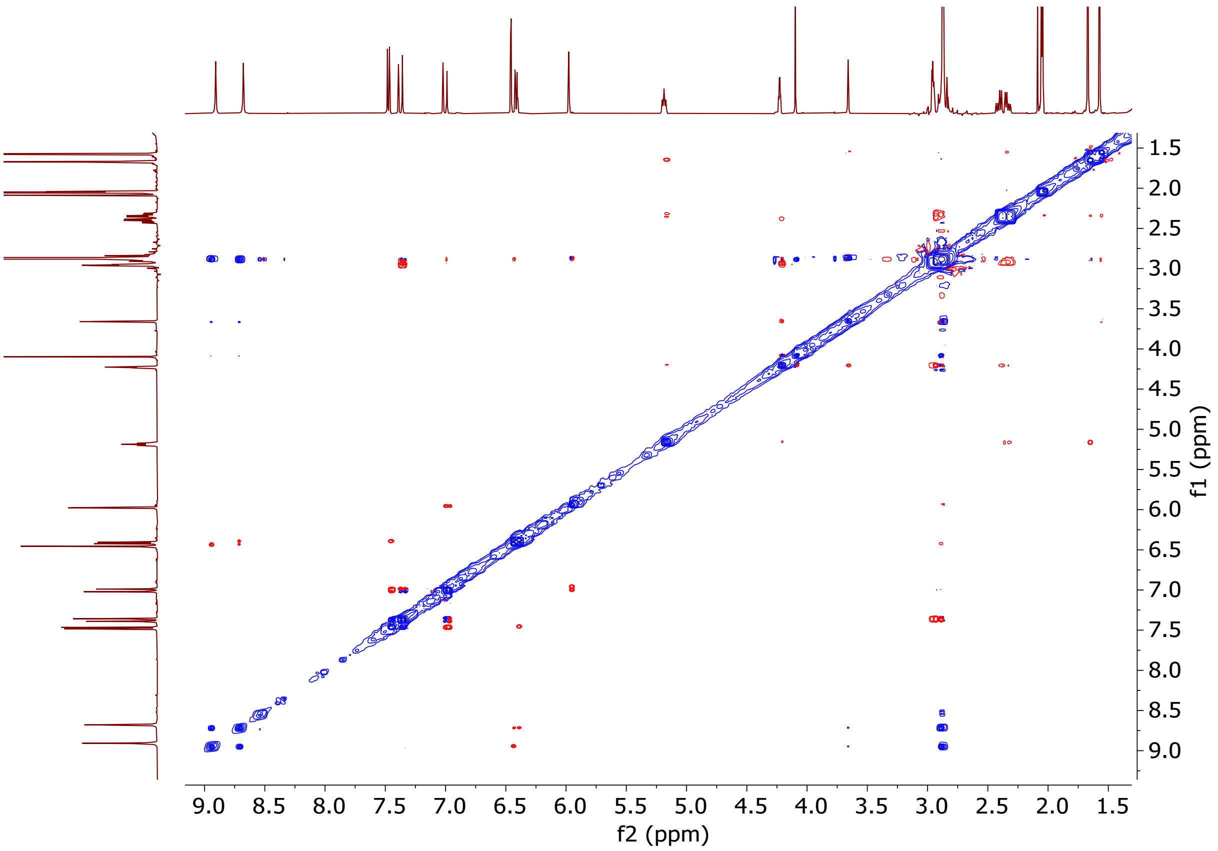

Figure S6. NOESY NMR spectrum of **1**.

**Sample Name**	DNT04	**Position**	P1-C9	**Instrument Name**	Instrument 1
**User Name**		**Inj Vol**	2	**InjPosition**	
**Sample Type**	Sample	**IRM Calibration Status**	Success	**Data Filename**	DNT04.d
**ACQ Method**	Cot ngan - MSMS_Pos.m	**Comment**		**Acquired Time**	01/07/2021 5:14:21 PM

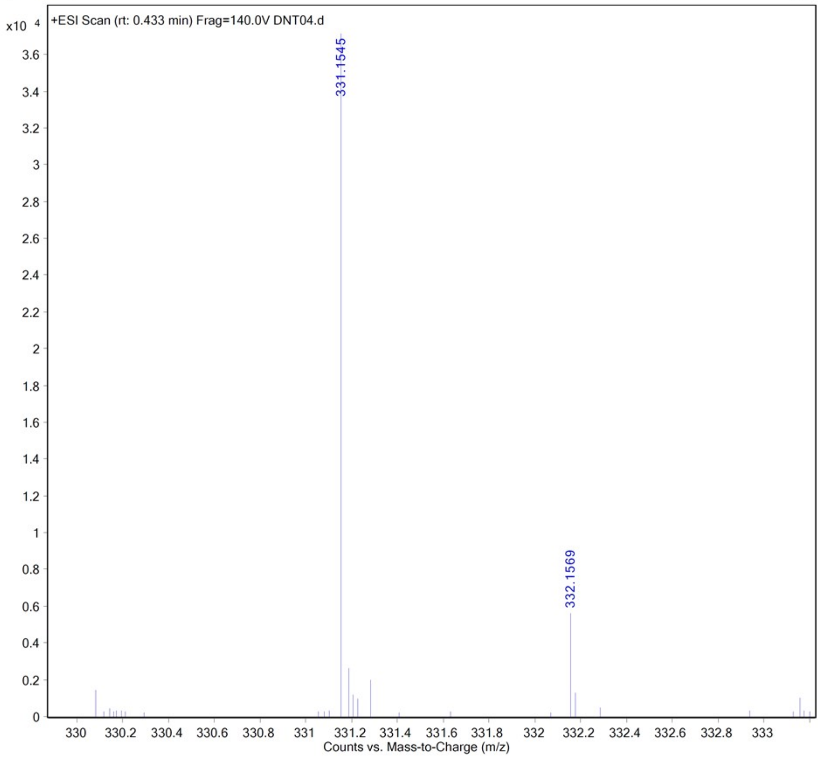

Figure S7. HRESIMS spectrum of **1**.

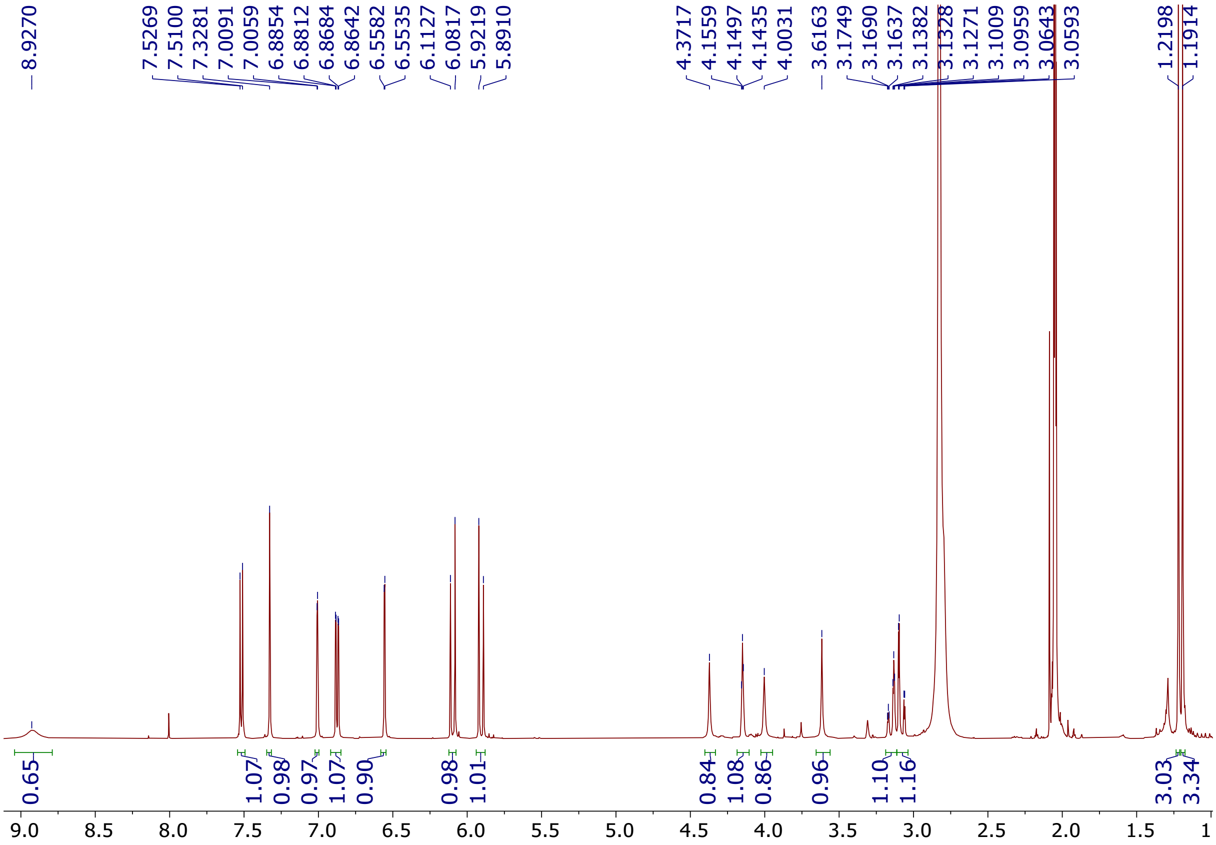

Figure S8. ^{1}H NMR spectrum of **2** (500 MHz, acetone-d_6).

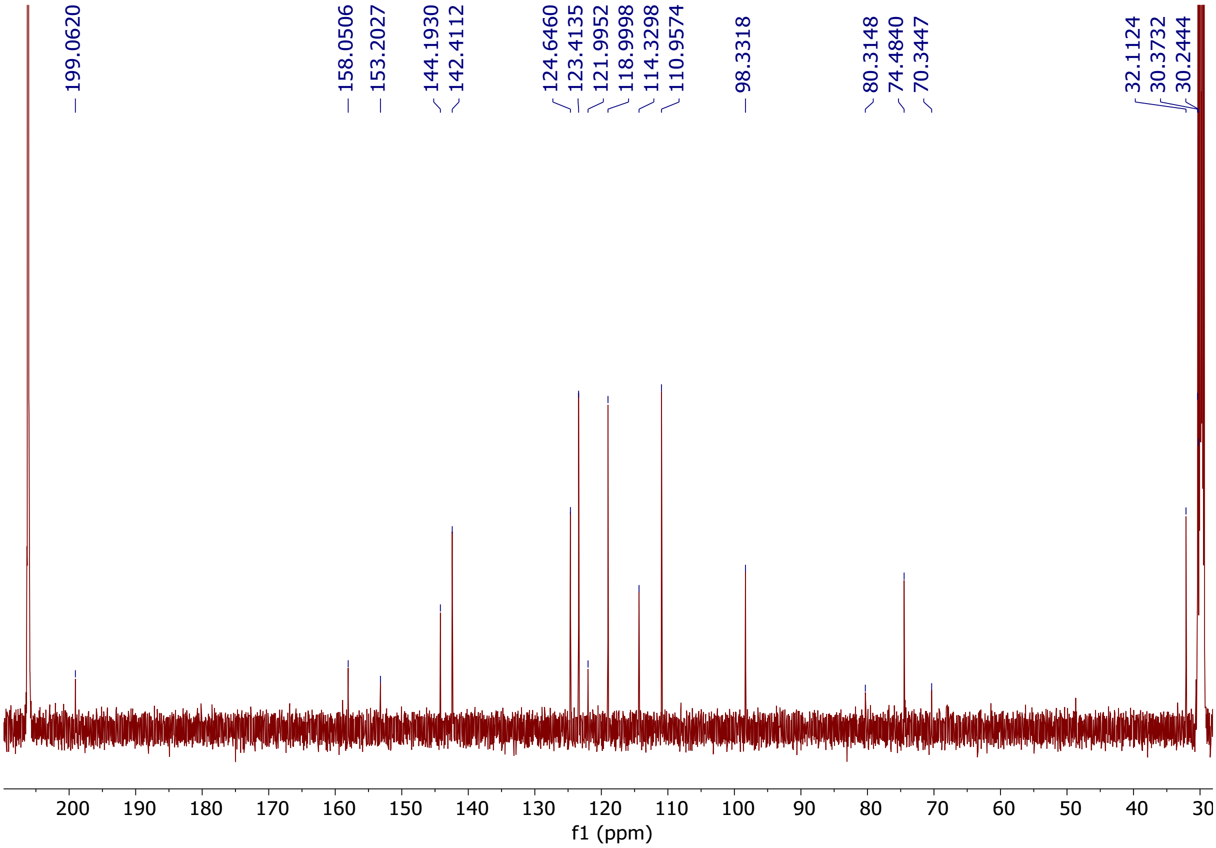

Figure S9. ^{13}C NMR spectrum of **2** (125 MHz, acetone-d_6).

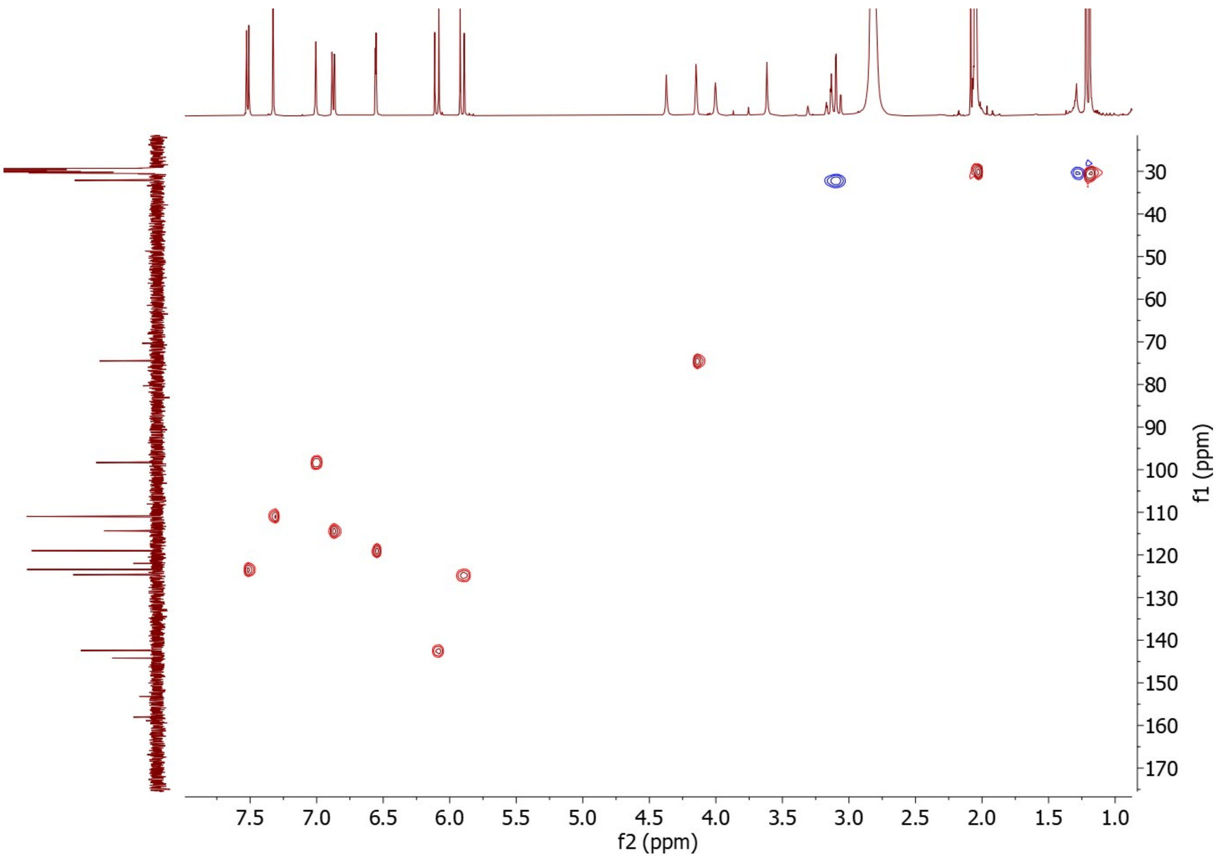

Figure S10. HSQC NMR spectrum of **2**.

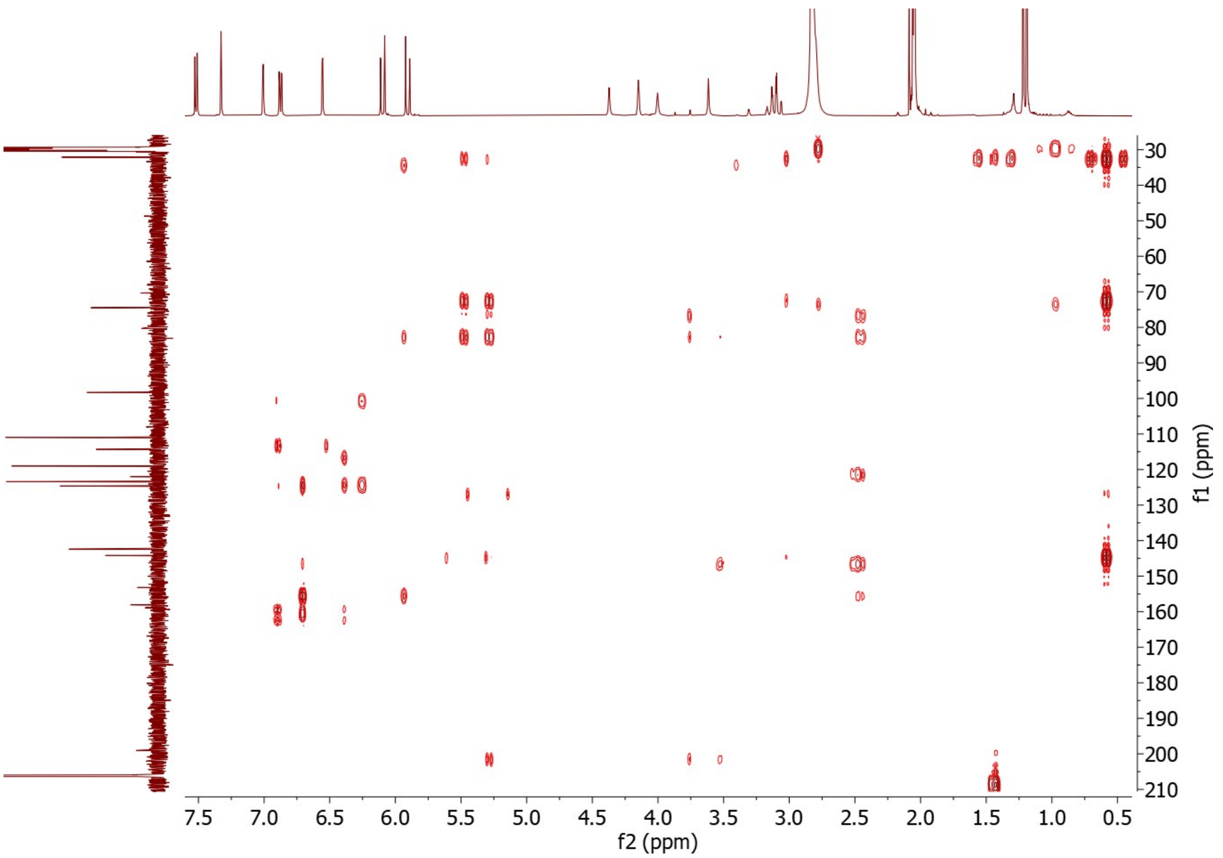

Figure S11. HMBC NMR spectrum of **2**.

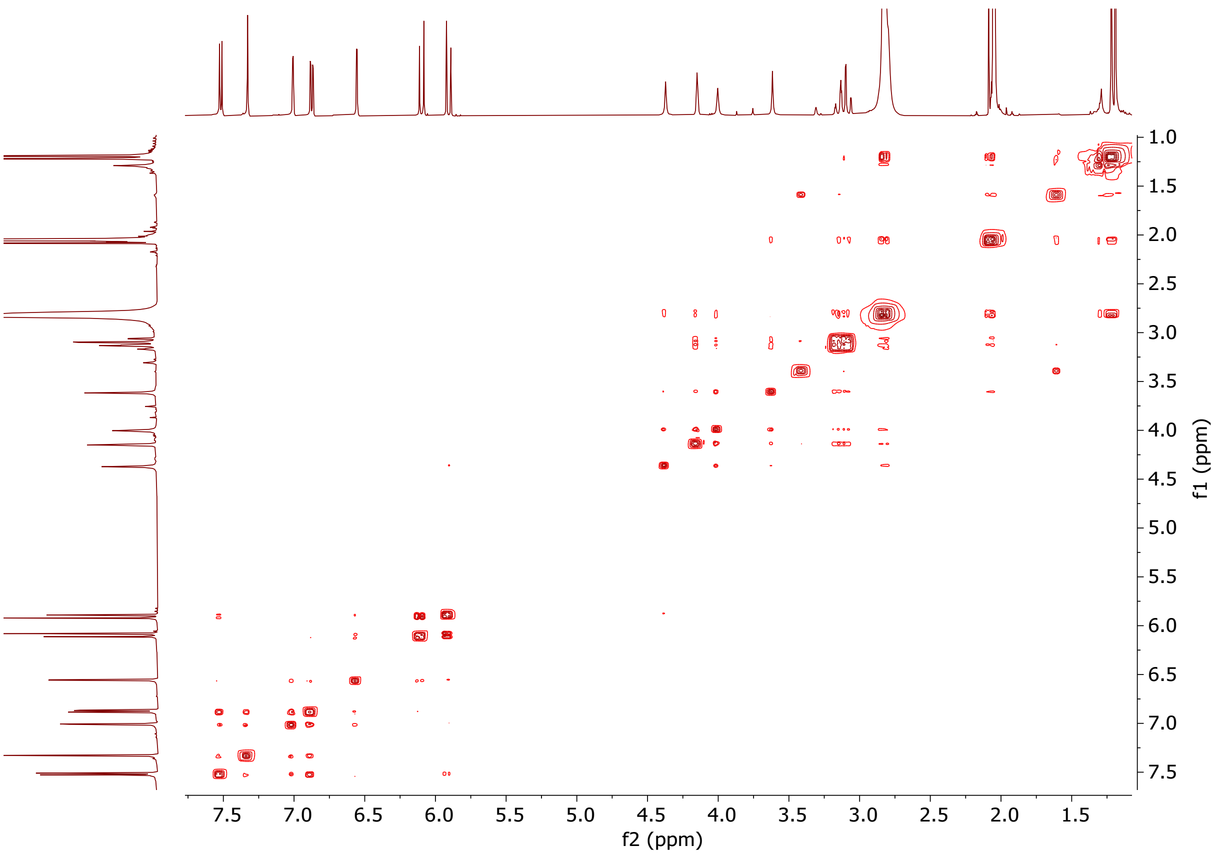

Figure S12. COSY NMR spectrum of **2**.

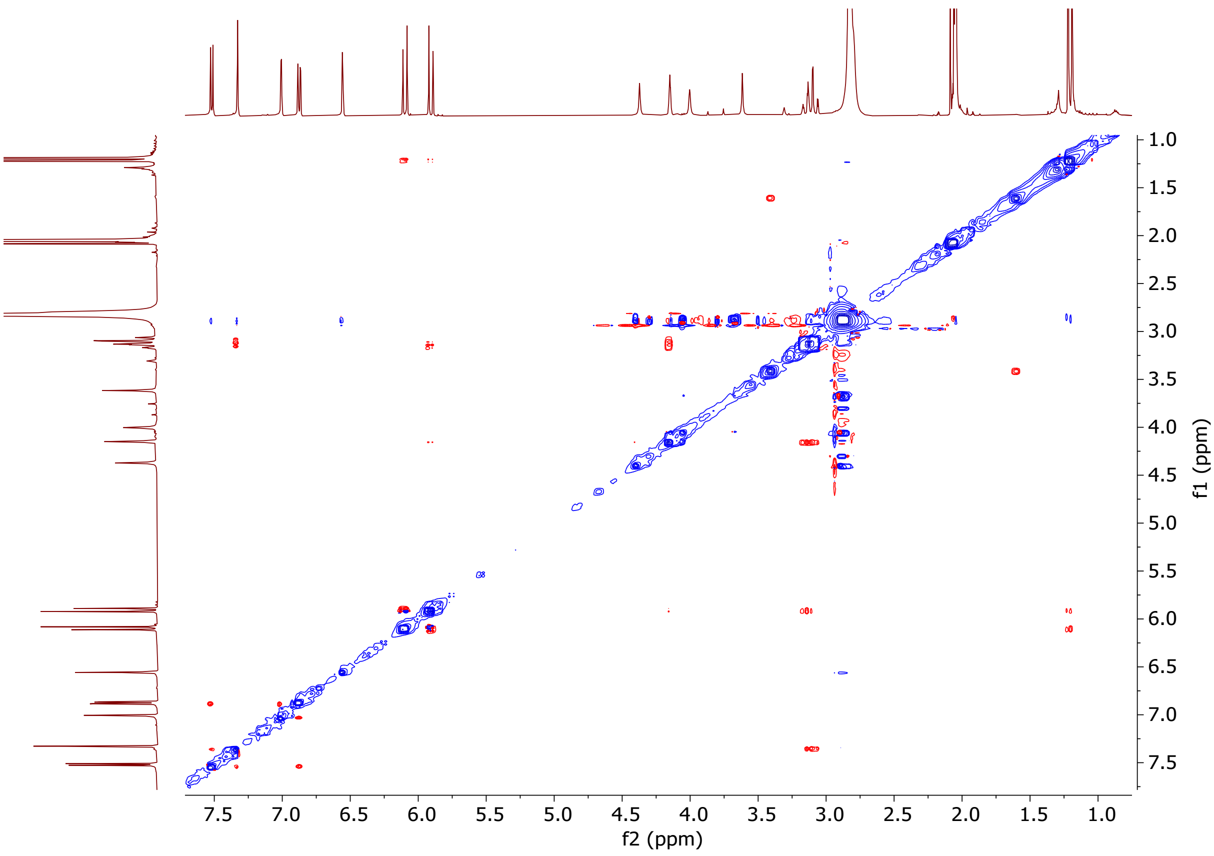

Figure S13. NOESY NMR spectrum of **2**.

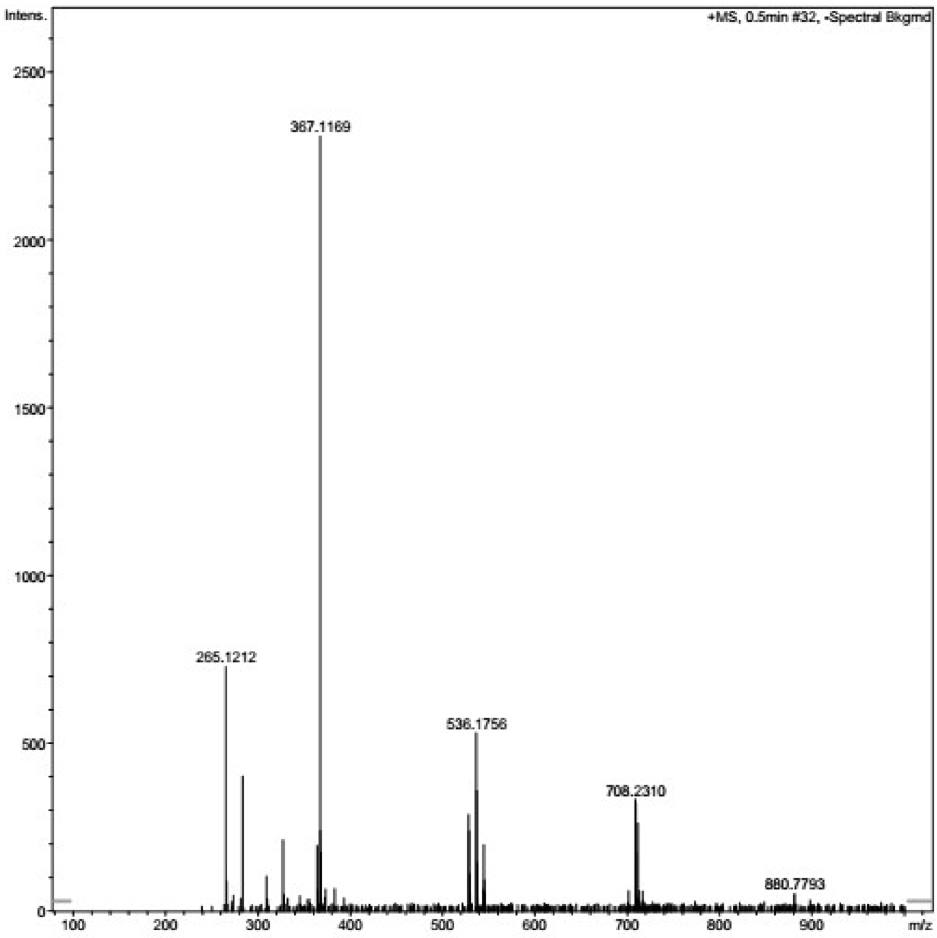

Figure S14. HRESIMS spectrum of **2**.

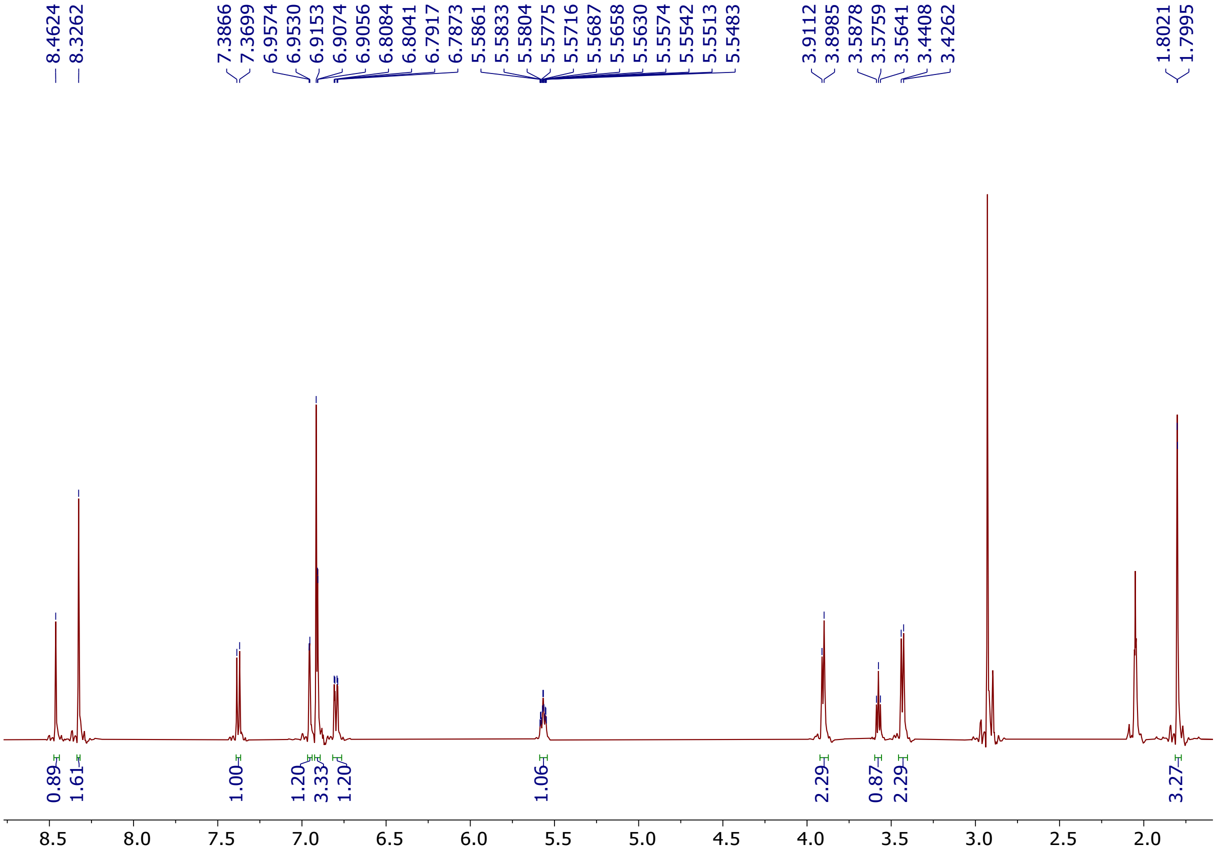

Figure S15. ^{1}H NMR spectrum of **3** (500 MHz, acetone-d_6).

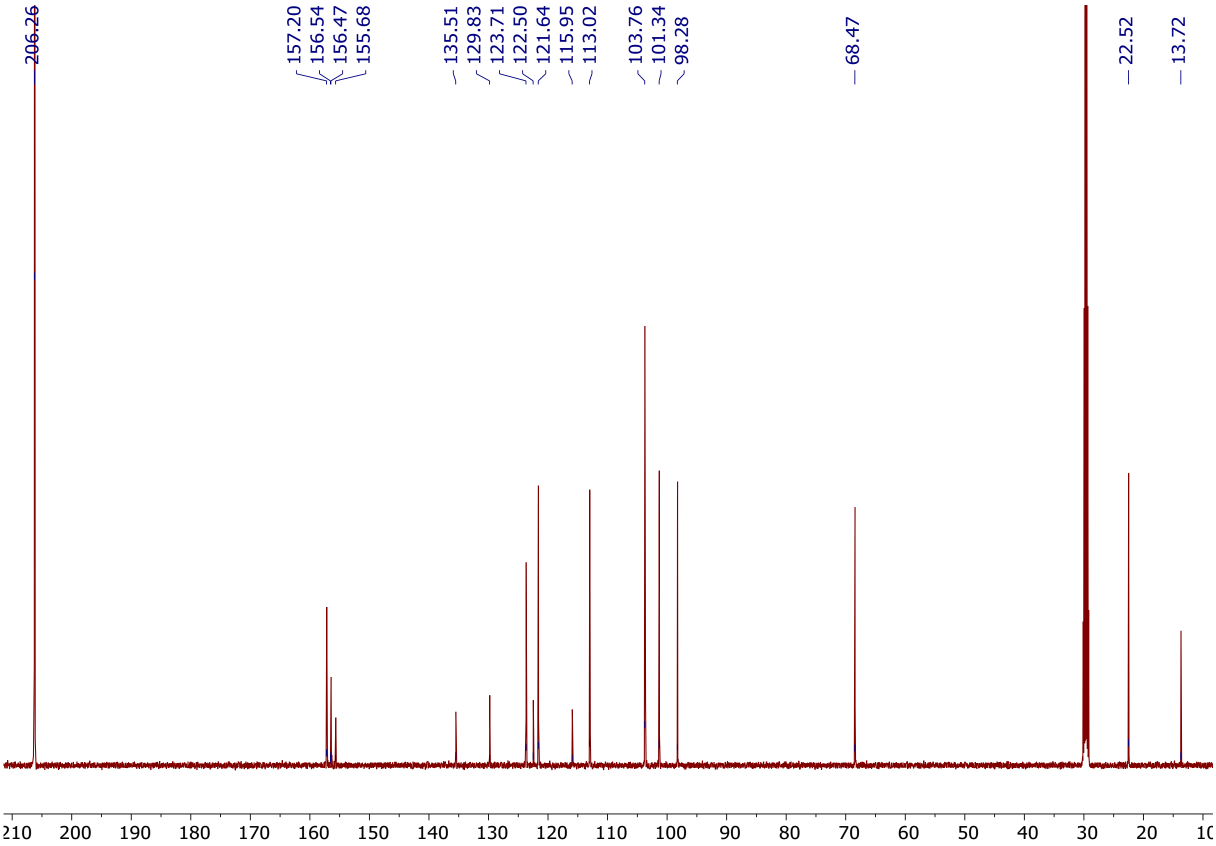

Figure S16. ^{13}C NMR spectrum of **3** (125 MHz, acetone-d_6).

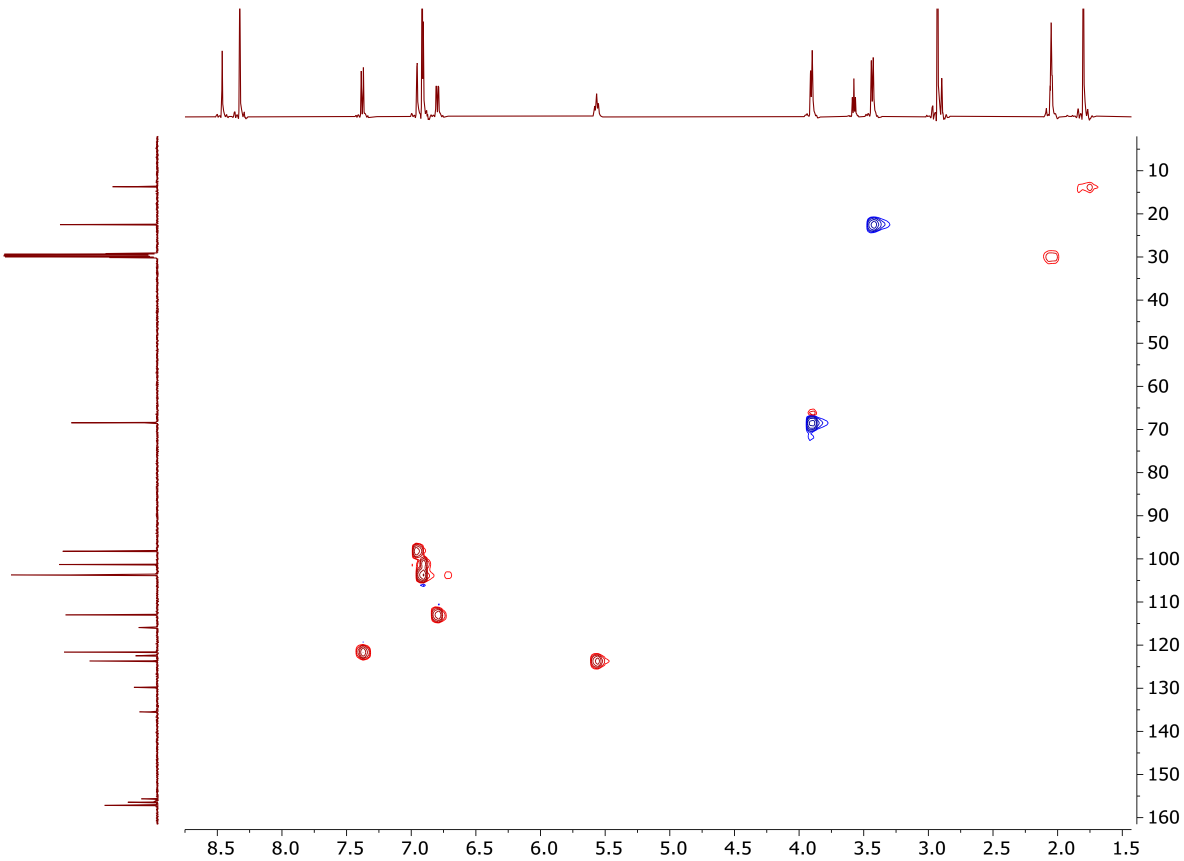

Figure S17. HSQC NMR spectrum of **3**.

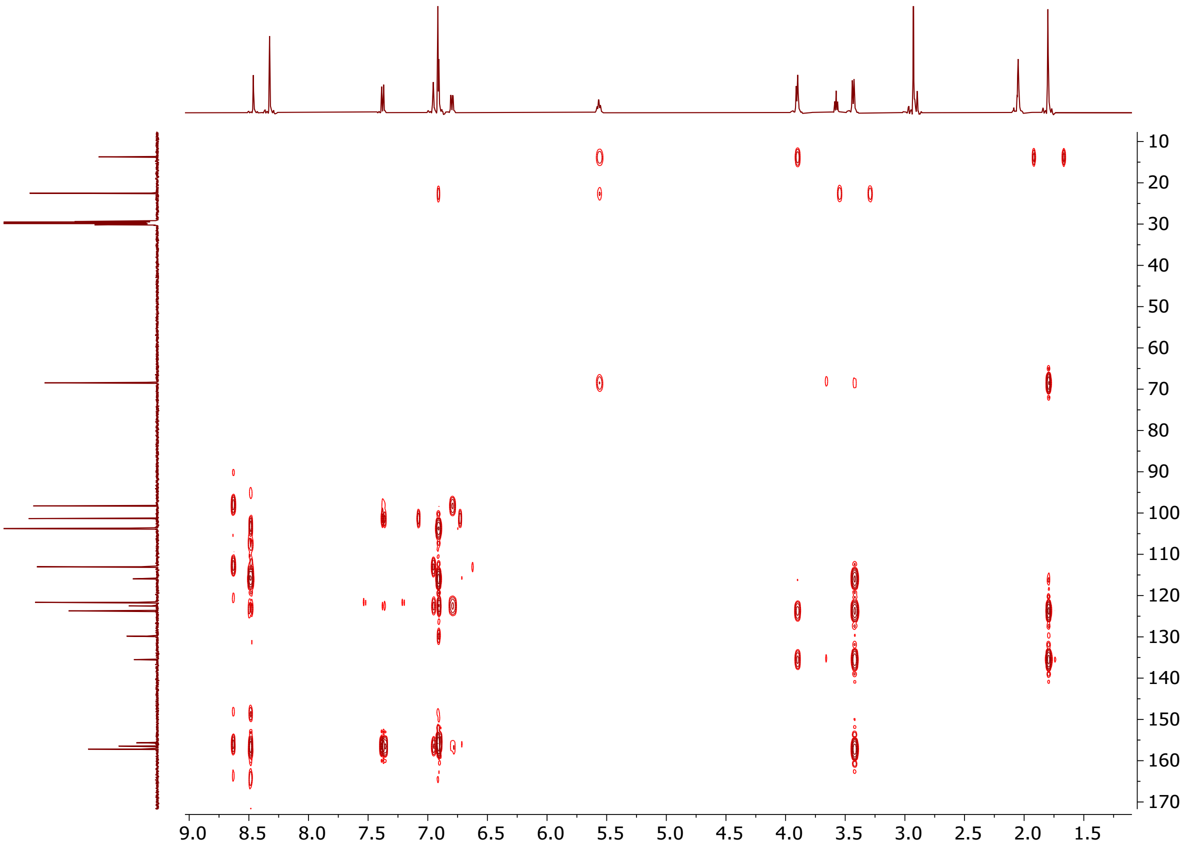

Figure S18. HMBC NMR spectrum of **3**.

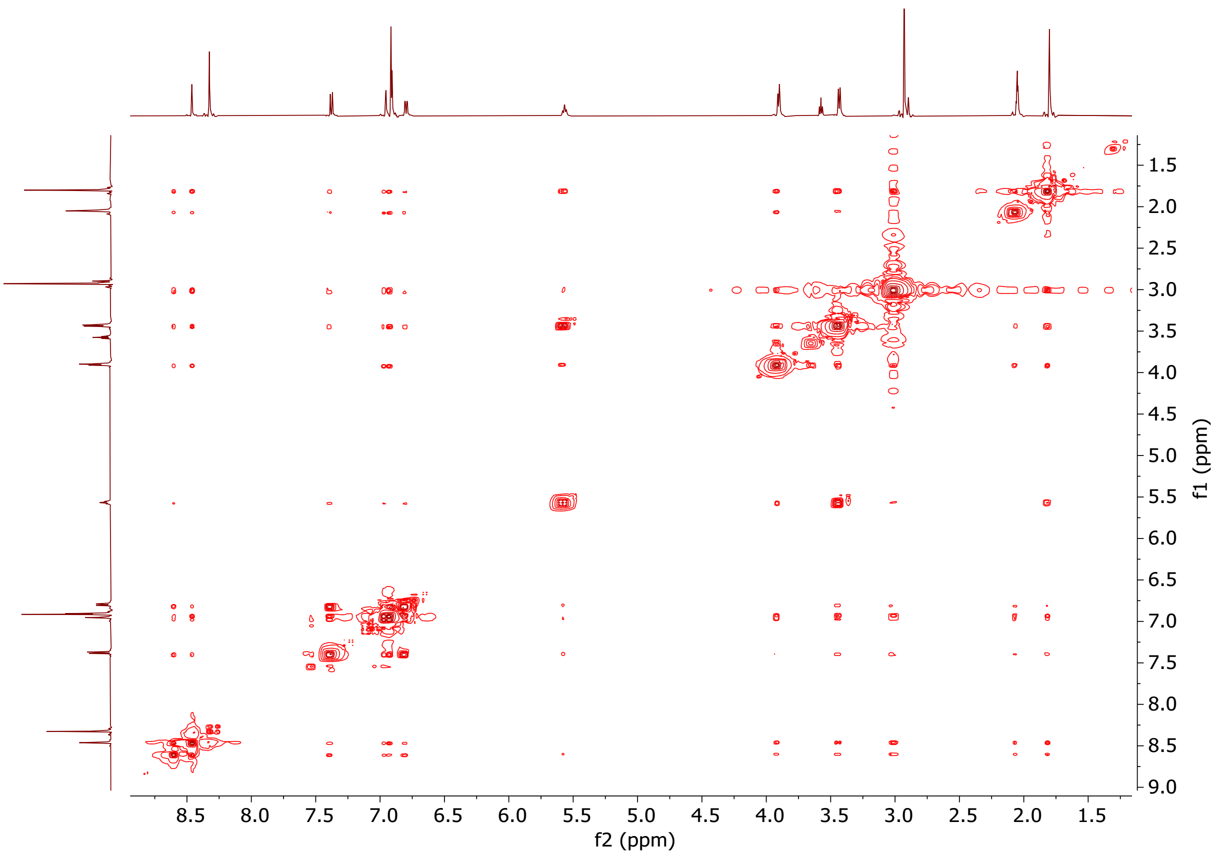

Figure S19. COSY NMR spectrum of **3**.

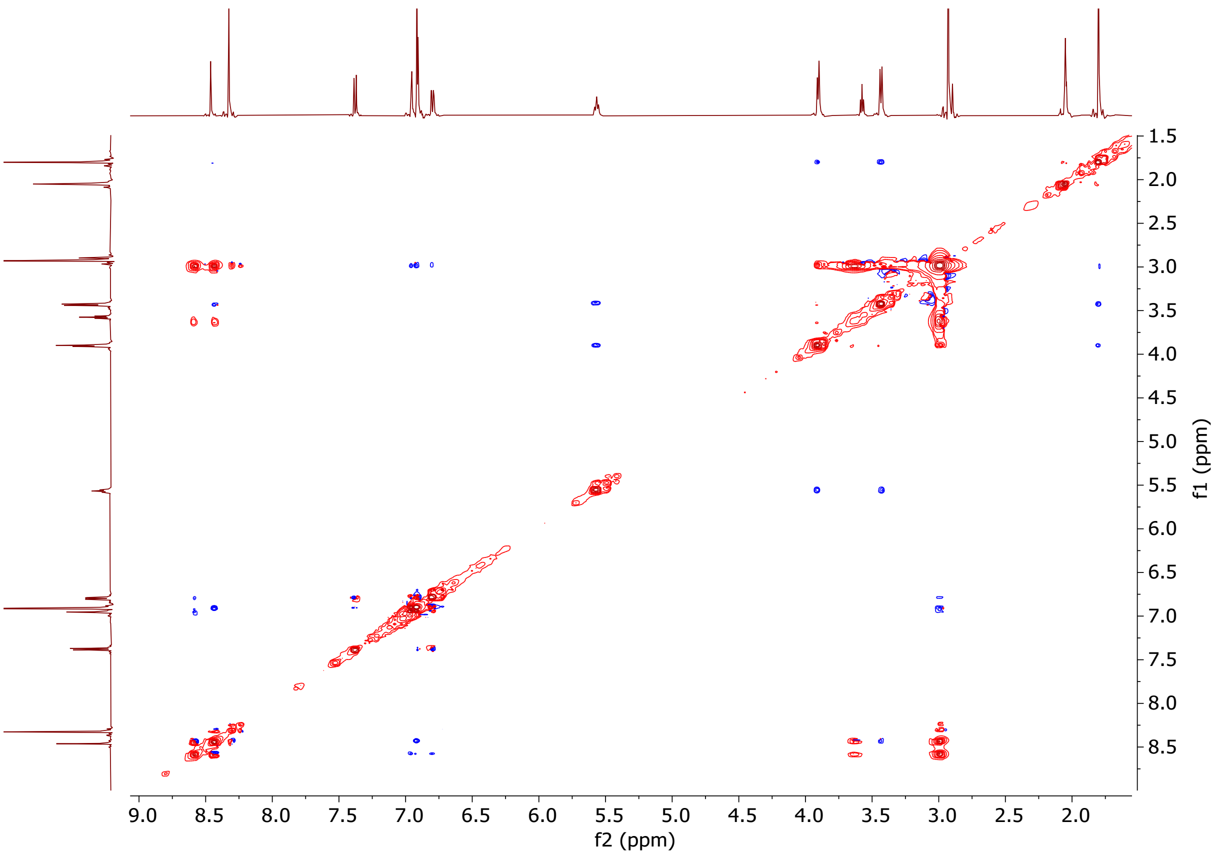

Figure S20. NOESY NMR spectrum of **3**.

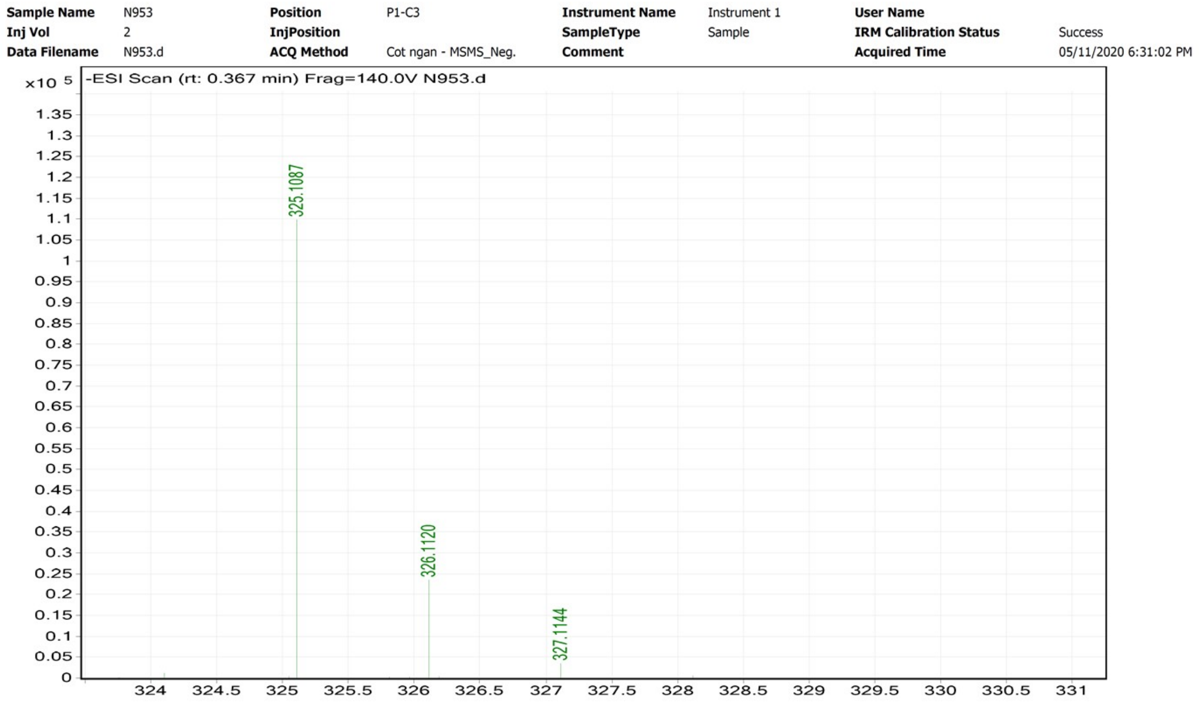

Figure S21. HRESIMS spectrum of **3**.

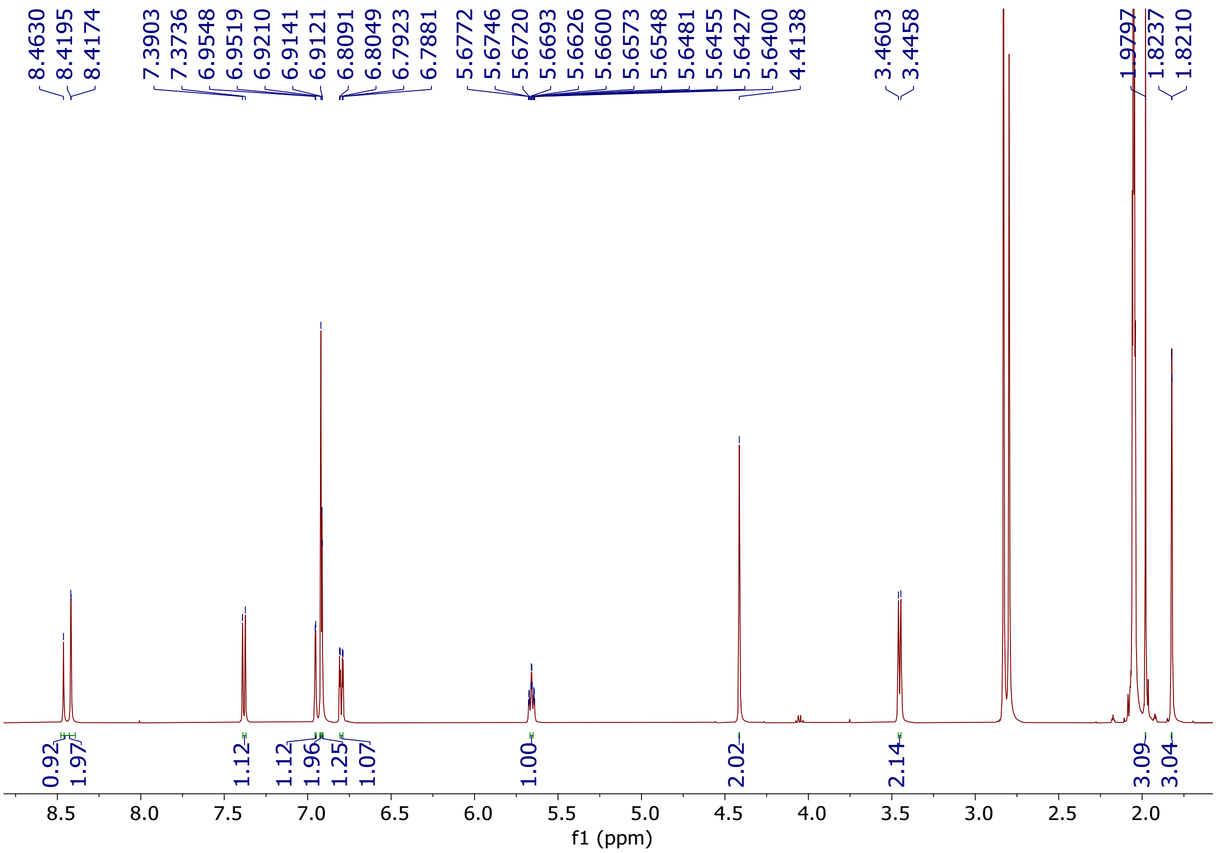

Figure S22. ^{1}H NMR spectrum of **4** (500 MHz, acetone-d_6).

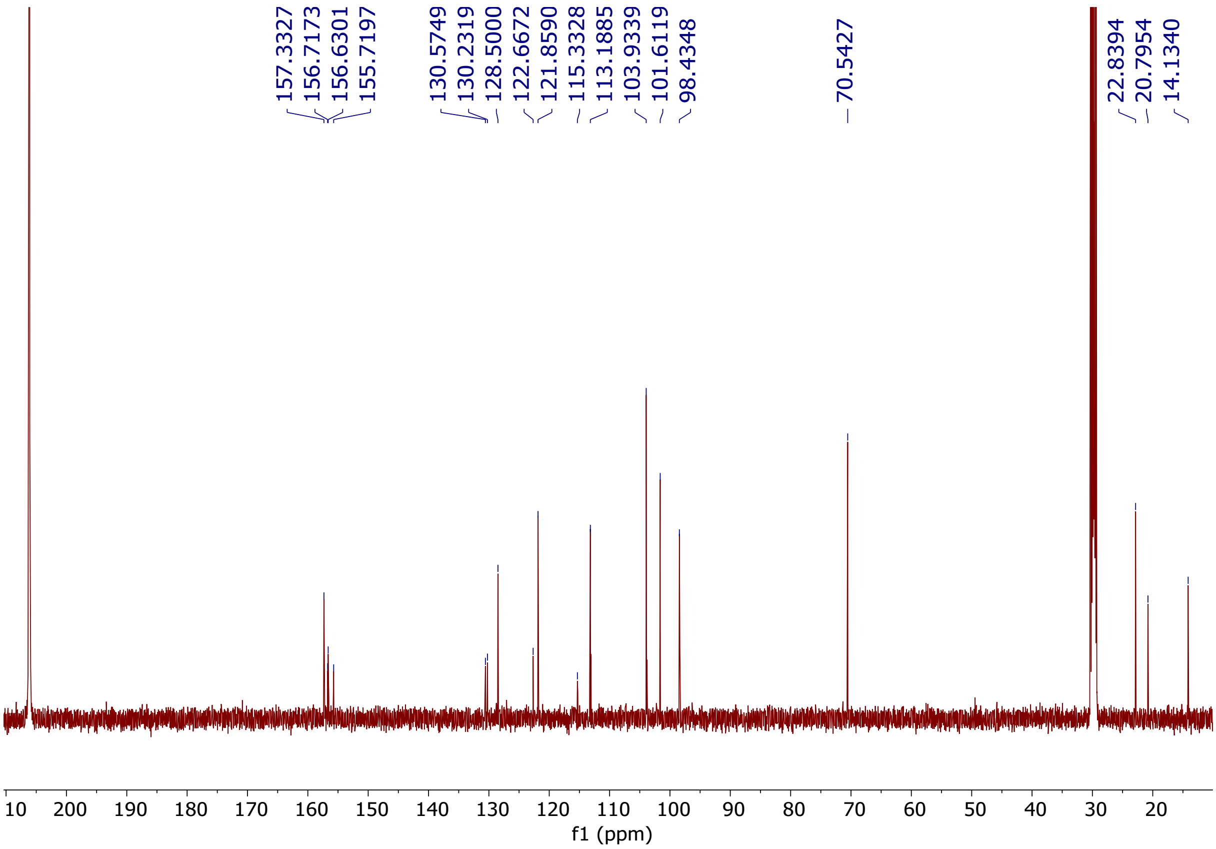

Figure S23. ^{13}C NMR spectrum of **4** (125 MHz, acetone-d_6).

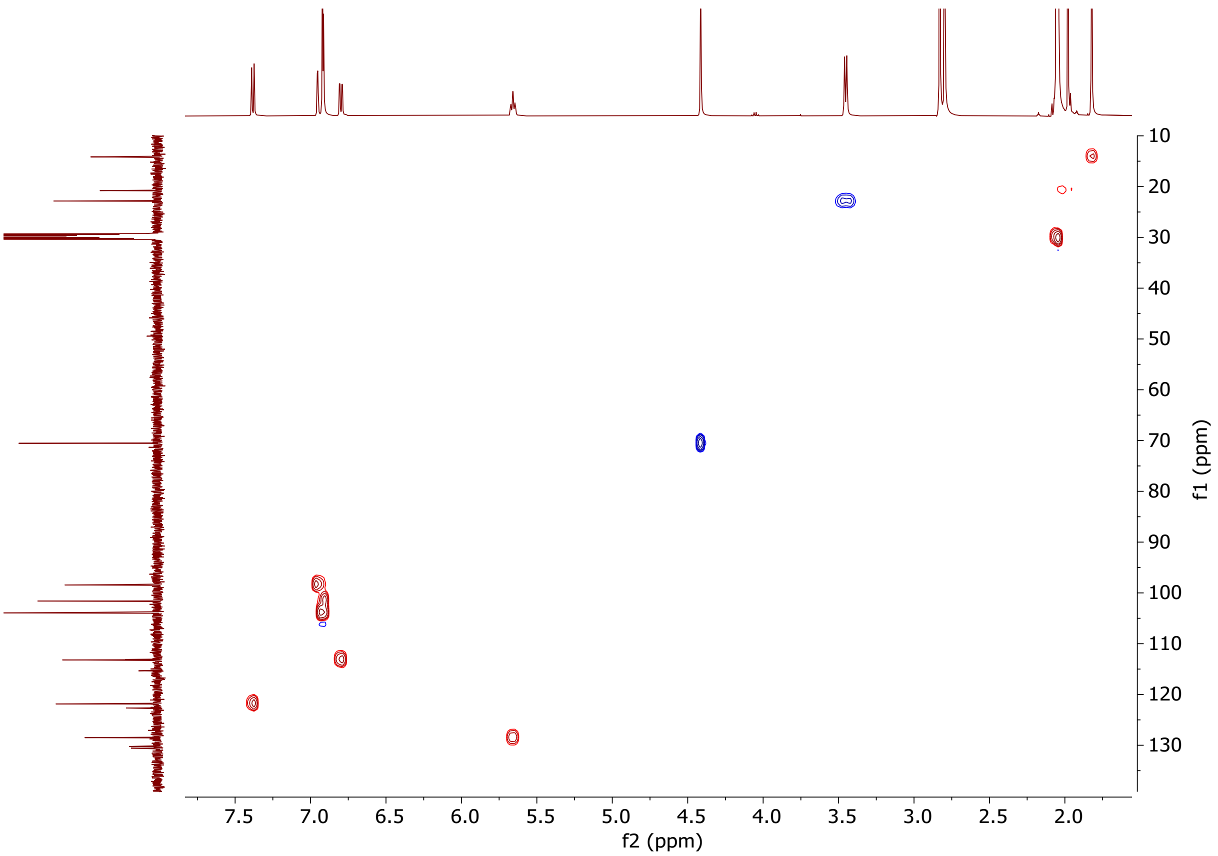

Figure S24. HSQC NMR spectrum of **4**.

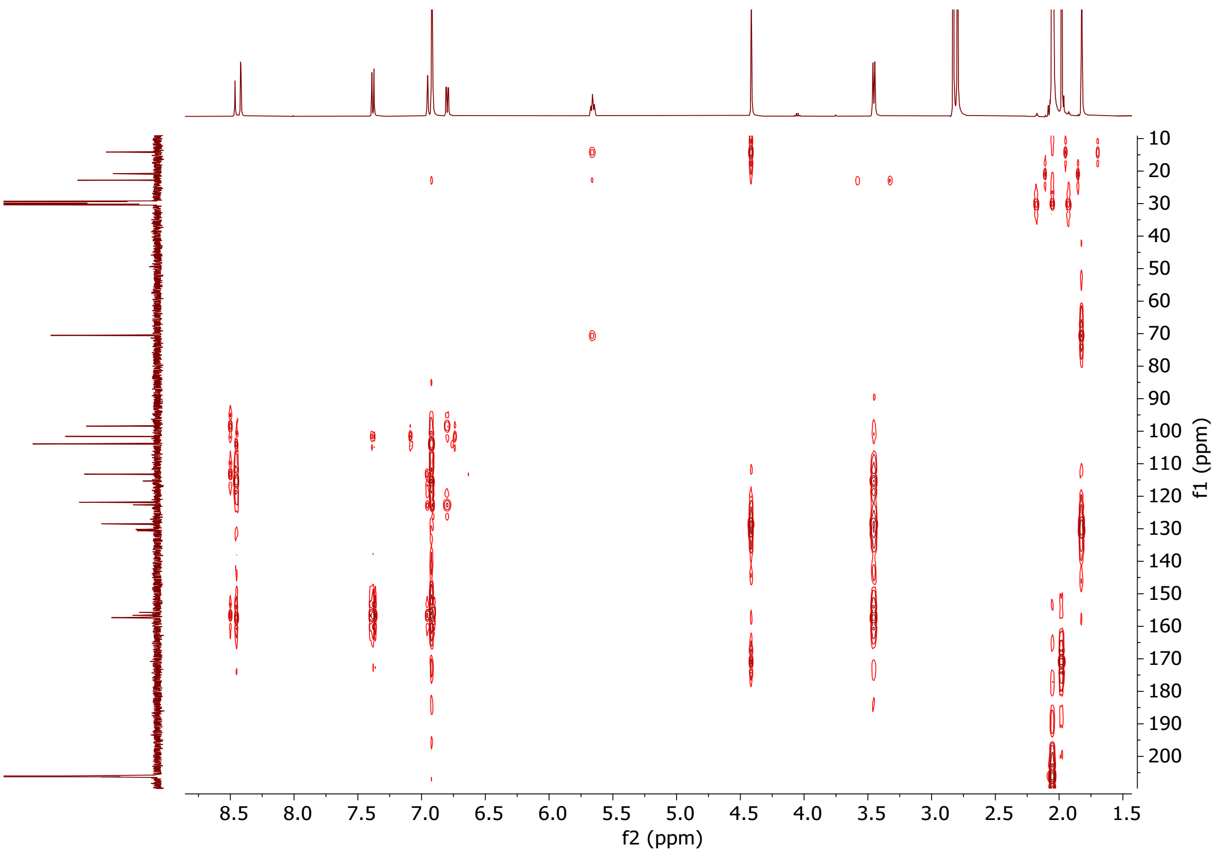

Figure S25. HMBC NMR spectrum of **4**.

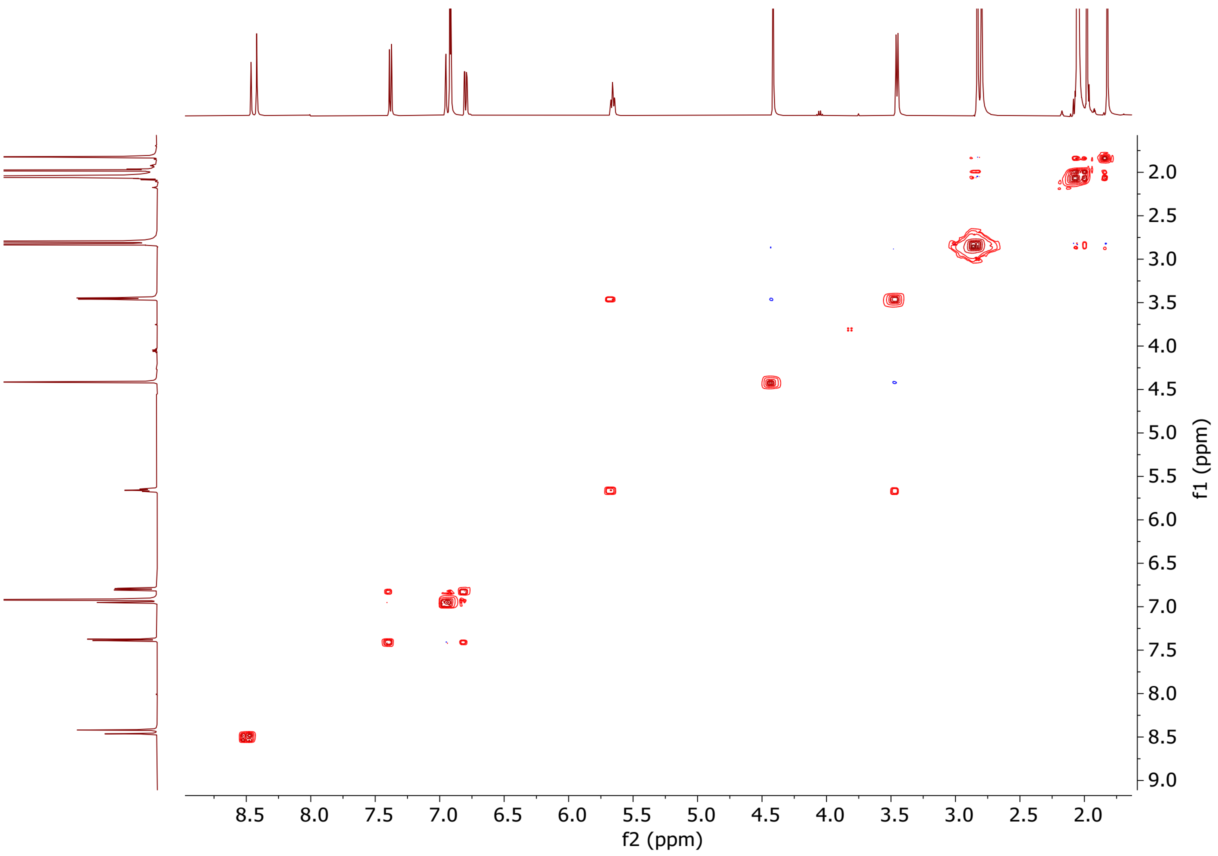

Figure S26. COSY NMR spectrum of **4**.

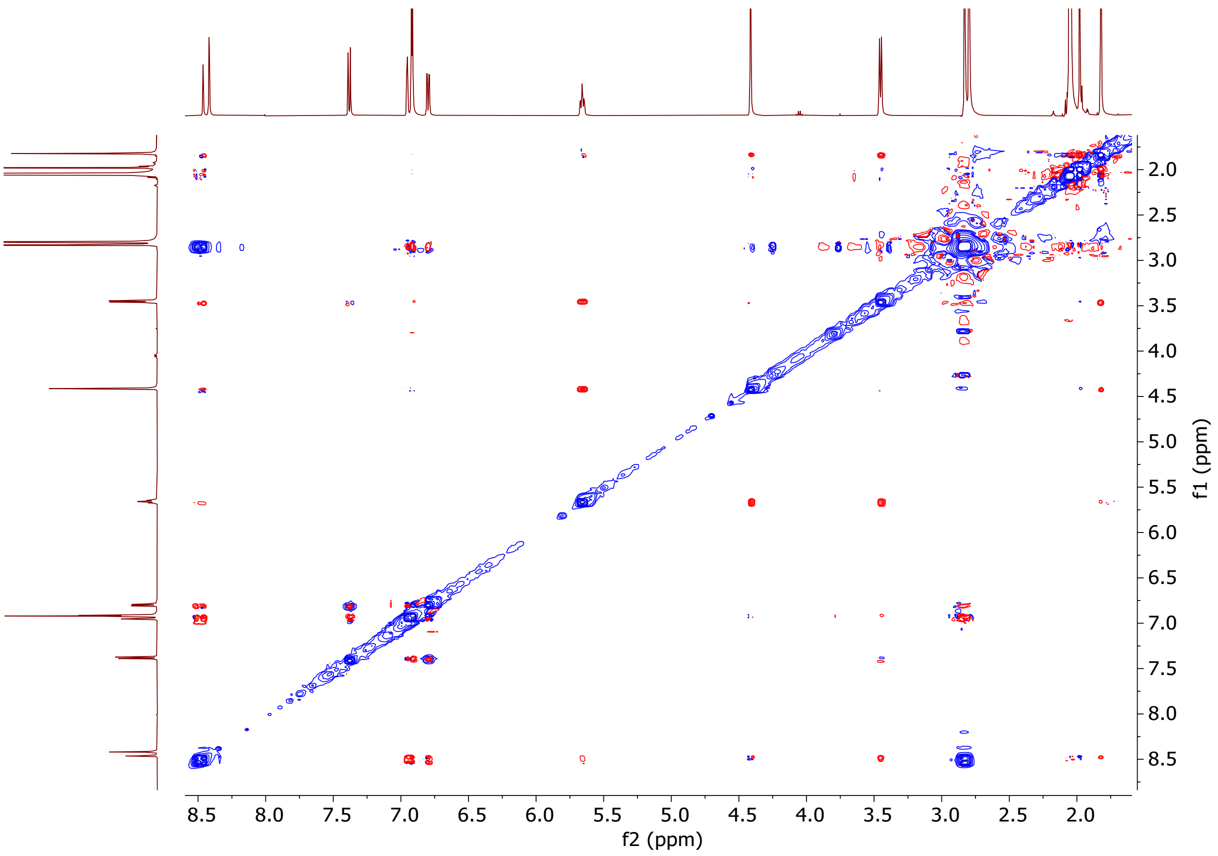

Figure S27. NOESY NMR spectrum of **4**.

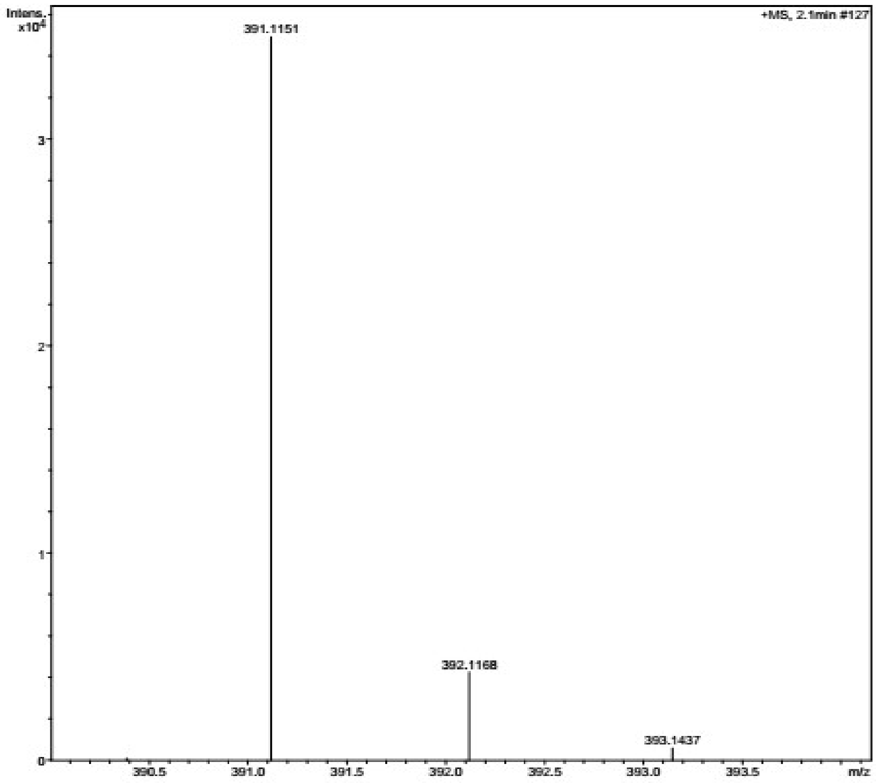

Figure S28. HRESIMS spectrum of **4**.
